# The centipede genus *Eupolybothrus* Verhoeff, 1907 (Chilopoda: Lithobiomorpha: Lithobiidae) in North Africa, a cybertaxonomic revision, with a key to all species in the genus and the first use of DNA barcoding for the group

**DOI:** 10.3897/zookeys.50.504

**Published:** 2010-06-30

**Authors:** Pavel Stoev, Nesrine Akkari, Marzio Zapparoli, David Porco, Henrik Enghoff, Gregory D. Edgecombe, Teodor Georgiev, Lyubomir Penev

**Affiliations:** 1 National Museum of Natural History, Tsar Osvoboditel Blvd. 1, 1000 Sofia, Bulgaria; 2 Research Unit of Biodiversity and Biology of Populations, Institut Superieur des Sciences Biologiques Appliquees de Tunis, 9 Avenue Dr. Zouheir Essafi, La Rabta, 1007 Tunis, Tunisia; 3 Universita degli Studi della Tuscia, Dipartimento di Protezione delle Piante, via S. Camillo de Lellis s.n.c., I-01100 Viterbo, Italy; 4 Biodiversity Institute of Ontario, University of Guelph, Guelph, Ontario N1G 2W1, Canada; 5 Natural History Museum of Denmark (Zoological Museum), University of Copenhagen, Universitetsparken 15, DK-2100 Kobenhavn O - Denmark; 6 The Natural History Museum, Department of Palaeontology, Cromwell Road, London SW7 5BD, UK; 7 Pensoft Publishers, 13a Geo Milev Str., 1111 Sofia, Bulgaria; 8 Bulgarian Academy of Sciences & Pensoft Publishers, 13a Geo Milev Str., Sofia, Bulgaria

**Keywords:** Eupolybothrus kahfi sp. n., Eupolybothrus nudicornis, North Africa, barcoding, cytochrome c oxidase I gene, troglomorphism, habitat preferences, interactive key, cybertaxonomy, semantic tagging, semantic enhancements

## Abstract

The centipede genus Eupolybothrus Verhoeff, 1907 in North Africa is revised. A new cavernicolous species, Eupolybothrus kahfi Stoev & Akkari, **sp. n.**, is described from a cave in Jebel Zaghouan, northeast Tunisia. Morphologically, it is most closely related to Eupolybothrus nudicornis (Gervais, 1837) from North Africa and Southwest Europe but can be readily distinguished by the long antennae and leg-pair 15, a conical dorso-median protuberance emerging from the posterior part of prefemur 15, and the shape of the male first genital sternite. Molecular sequence data from the cytochrome c oxidase I gene (mtDNA–5’ COI-barcoding fragment) exhibit 19.19% divergence between Eupolybothrus kahfi and Eupolybothrus nudicornis, an interspecific value comparable to those observed among four other species of Eupolybothrus which, combined with a low intraspecific divergence (0.3–1.14%), supports the morphological diagnosis of Eupolybothrus kahfi as a separate species. This is the first troglomorphic myriapod to be found in Tunisia, and the second troglomorph lithobiomorph centipede known from North Africa. Eupolybothrus nudicornis is redescribed based on abundant material from Tunisia and its post-embryonic development, distribution and habitat preferences recorded. Eupolybothrus cloudsley-thompsoni Turk, 1955, a nominal species based on Tunisian type material, is placed in synonymy with Eupolybothrus nudicornis. To comply with the latest technological developments in publishing of biological information, the paper implements new approaches in cybertaxonomy, such as fine granularity XML tagging validated against the NLM DTD TaxPub for PubMedCentral and dissemination in XML to various aggregators (GBIF, EOL, Wikipedia), vizualisation of all taxa mentioned in the text via the dynamically created Pensoft Taxon Profile (PTP) page, data publishing, georeferencing of all localities via Google Earth, and ZooBank, GenBank and MorphBank registration of datasets. An interactive key to all valid species of Eupolybothrus is made with DELTA software.

## Introduction

The lithobiid subfamily Ethopolyinae is represented in Europe and Africa by a single genus, Eupolybothrus Verhoeff, 1907, which currently comprises around 20 valid species as well as a few poorly known species and subspecies, collectively arranged in seven subgenera (Zapparoli 2003, Minelli 2006). The genus ranges from Central and South Europe to the Middle East and Maghreb with highest species diversity in the Appenine and Balkan peninsulas (Zapparoli 2003). In North Africa, Eupolybothrus is known only from a single, quite ubiquitous species, Eupolybothrus nudicornis (Gervais, 1837), the range of which extends from northern Morocco to northwestern Libya. It also occurs in France, mainland Spain and Italy, as well as in several West Mediterranean islands (Manfredi 1939, Minelli 2006).

The identity of Eupolybothrus nudicornis has been a subject of controversy for more than a century. The polymorphic external anatomy shown by the species throughout its broad geographic range led to the description of several morphologically similar taxa that were sometimes based only on a single type specimen (Minelli 2006). Currently the list of named taxa allied to Eupolybothrus nudicornis comprises six species and eight subspecies/varieties described from different Mediterranean countries: Eupolybothrus koenigi (Verhoeff, 1891), Eupolybothrus lebruni (Dobroruka, 1968), Eupolybothrus monilicornis (Newport, 1849), Eupolybothrus elongatus alpinus (Brolemann, 1930), Eupolybothrus elongatus aprutianus (Manfredi, 1950), Eupolybothrus elongatus calabrus (Manfredi, 1933), Eupolybothrus elongatus imperanus (Verhoeff, 1937), Eupolybothrus elongatus levis (Verhoeff, 1943), Eupolybothrus elongatus oraniensis (Verhoeff, 1901), Eupolybothrus elongatus sardus (Manfredi, 1956), Eupolybothrus impressus corsicus (Brölemann, 1903), Eupolybothrus cloudsley-thompsoni Turk, 1955, Eupolybothrus osellai Matic, Floca & Hurezeanu, 1992, and Eupolybothrus ruffoi Matic, Floca & Hurezeanu, 1992. The list has been reduced through the course of taxonomic revisions, with most of the names eventually being considered synonyms of Eupolybothrus nudicornis. Currently the taxonomic position of three of them, Eupolybothrus cloudsley-thompsoni from Tunisia, Eupolybothrus osellai and Eupolybothrus ruffoi, both from Italy, is uncertain and needs reappraisal (Minelli 2006). A more comprehensive study of the whole group has never been undertaken and it is likely that some synonyms will turn out to be valid species after contemporary morphological and molecular methods are applied.

The aim of present paper is to put on record all North African material of Eupolybothrus amassed during recent years and also found in old collections of different European museums. We redescribe Eupolybothrus nudicornis and describe a new species discovered in a cave in Tunisia. The new species is distinguished from the nearest congener morphologically as well as using the cytochrome c oxidase I gene (mtDNA–5’ COI-barcoding fragment). We also discuss the morphological variability and post-embryonic development of Eupolybothrus nudicornis and provide an overview of its habitat preferences and distribution in Africa. We outline some of the existing taxonomic problems in the genus Eupolybothrus and provide a key to all currently valid species of the genus.

### Historical account.

The earliest record of the genus Eupolybothrus in North Africa was made by Carl Ludwig Koch (1841), who described Lithobius impressus from Alger and Oran in Algeria. A few years later, Lucas (1849) recorded the same species from other localities in the country: Lac Tonga and Houbeira, La Calle, Constantine, Bône, and Philippeville. Newport (in Lucas 1849) described two further species from Algeria, Lithobius monilicornis from Boudjaréa near Alger and Lithobius elongatus from Lac Tonga, Houbeira and La Calle. The former was tentatively synonymised with Lithobius impressus by Meinert (1872), who redescribed the species based on new material from Algeria. Eason (1972a) confirmed Meinert’s synonymy and suggested elongatus as a possibly good subspecies of Eupolybothrus impressus.

Verhoeff (1891) described Lithobius koenigi from Tunis and ten years later Lithobius elongatus oraniensis from “Rio Salado, Chabael Ham, Djebel el Tessala, Saida and Hammam Bou Hadjar” (Verhoeff 1901)), all in Algeria. Subsequently, Lithobius koenigi was synonymised with Lithobius elongatus by Silvestri (1896) who recorded it also from Tunis, Carthage, Souk el Arba (now Jendouba), Aïn Draham, Babouch and Tabarka. Eason (1972a) proposed the synonymy of Eupolybothrus elongatus oraniensis with Eupolybothrus impressus.

In 1892 Pocock recorded Lithobius impressus from Kherrarta, Alger, Constantine and Hammam Ri’irha in Algeria, and from Tunis in Tunisia (Pocock 1892). Attems (1908) recorded Polybothrus koenigi from Aïn Draham in Kroumirie, Tunisia. In the checklist of North African myriapods Brolemann (1921) mentioned Bothropolys impressus for Tunisia, Algeria and Morocco, referring to it most of the previous records from North Africa (those of e.g., C.L. Koch 1841, Lucas 1849, Verhoeff 1891, Pocock 1892, Silvestri 1896, Attems 1908), and also questioned the occurrence of Bothropolys monilicornis and Lithobius elongatus in Algeria, and that of Lithobius nudicornis in Tunisia and Algeria. Subsequently, having material from Tipasa and Djebel Bou Zegza in Algeria, he revived koenigi as a variety of Bothropolys elongatus distinguishing it by the spinulation of leg-pairs 14 and 15 and the more slender prefemora of leg-pair 15 (Brolemann 1925, 1931a). Only these two taxa were included in his identification key to the North African centipedes (Brolemann 1932).

Silvestri (1897) was first to draw attention to Lithobius nudicornis from Sicily being conspecific with Eupolybothrus impressus. Although this fact has been commented upon by several authors (e.g., Jeekel 1967, Eason 1972b) it was Minelli (1983a) who quite recently validated the name. Minelli (1983a) wrote “Eason (1980) refers to this Sardinian Eupolybothrus under the name Eu. impressus (C.L. Koch); however, it is probable that a single species should be recognized in the complex nudicornis – impressus – elongatus, with nudicornis as senior synonym. In any case, I agree with Eason in so far I recognize a single taxon of this group in Sardinia, despite some habitus differences between different populations”. All subsequent taxonomic publications where Eupolybothrus nudicornis is mentioned refer to it as a full species and do not recognize the existence of subspecies (e.g., Minelli 1983b; Foddai et al. 1995; Zapparoli 1994, 1995a, 2006, 2009; Zapparoli et al. 2004; Minelli 2006; Iorio 2008a,b).

The first and hitherto only record of the genus in Libya comes from Manfredi (1939) who recorded Bothropolys elongatus from Bu Gheilan (Tripolitania). Turk (1955) described a new species, Eupolybothrus cloudsley-thompsoni, collected near a Roman aqueduct south of Tunis. Dobroruka (1968) described another species, Schizopolybothrus lebruni, from Djebel Mansour (Pont du Fahs) also in Tunisia. Zapparoli (1985) synonymised Dobroruka’s species with Eupolybothrus impressus elongatus and suggested that Eupolybothrus cloudsley-thompsoni could also be its synonym but type specimens needed to be examined. Having on disposal specimens from Morocco (Rif), Algeria (Djudjura Mts.) and Tunisia (Thala, Nabeul), he confirmed the earlier observation of Eason (1972a) that in North Africa the species is represented by two subspecies – Eupolybothrus impressus impressus and Eupolybothrus impressus elongatus, which can be readily distinguished by the tarsal spinulation of the penultimate and ultimate pairs of legs and by the shape of tergite 9. More recently, Zapparoli (1995a) recorded Eupolybothrus nudicornis from the Italian islands Lampedusa and Pantelleria, which are situated close to the Tunisian coast.

Verhoeff (1907) split the genus Polybothrus into three subgenera, namely Allopolybothrus, Propolybothrus and Eupolybothrus. Crabill (1955) and Jeekel (1963) showed that they were validly proposed, opposing the opinion of Chamberlin (1925), as Jeekel (1963) designated Lithobius koenigi as the type species of subgenus Allopolybothrus. Subsequently, Jeekel (1967) reviewed the genus Eupolybothrus and resolved several nomenclatorial problems, also providing a list of all taxa assigned to the genus to that time. CHILOBASE (Minelli 2006) is a contemporary web-based database of centipede names and lists all currently valid species in the genus, nominal subgenera (see Jeekel 1963), and global species distributions.

## Material and methods

### Collections.

Unless stated otherwise, the material treated herein has been collected from Tunisia in March 2008 and March 2009 by N. Akkari, P. Stoev and H. Enghoff, and also in the course of individual excursions of N. Akkari to different regions of the country in the period 2003-2008. The material is preserved in 70% or 96% ethanol and is shared between the Field Museum of Natural History, Chicago (FMNH), National Museum of Natural History, Sofia (NMNHS), Natural History Museum of Denmark, Copenhagen (ZMUC) and Biodiversity Institute of Ontario, Guelph (BIO). Additional type and non-type specimens of Eupolybothrus from North Africa housed in the Hungarian Natural History Museum (HNHM), the Natural History Museum, London (NHM), ZMUC and the private collection of Marzio Zapparoli (CMZ) were also incorporated in the present study. Photos were taken mainly with a Leica DFC 420 digital camera mounted on a Leica MZ16A stereomicroscope, and were processed using the program Automontage Pro software (Syncroscopy, Cambridge, UK) for image-stacking 3D focus expansion. Terminology for external anatomy follows Bonato et al. (in prep.).

### Molecular methods.

Eleven specimens from 5 species were used for genetic examination of the divergence among species of the genus. Ten specimens that sample 4 species were barcoded in the context of a global campaign on Myriapoda initiated as a part of the ‘Barcode of Life’ project (iBOL WorkGroup 1.9 ‘Terrestrial surveillance’) (Appendix C doi:10.3897/zookeys.50.504-app.C). To this dataset we added a sequence from GenBank for a fifth species, Eupolybothrus fasciatus (AY214420) (Edgecombe and Giribet 2004). Sequences are publicly available on BOLD (Ratnasingham and Hebert 2007; http://www.barcodinglife.org) within the project PSEKA and in GenBank (accession numbers in Table 6).

Lysis of the tissues was carried out in 50 µl volume of lysis buffer and proteinase K incubated at 56°C overnight. DNA extraction followed a standard automated protocol on 96-well glass fibre plates (Ivanova et al. 2006). The 5’ region of COI used as a standard DNA barcode was amplified using M13 tailed primers LCO1490 and HCO2198 (Folmer et al. 1994). A standard PCR reaction protocol was used for PCR amplifications and products were checked on a 2% E-gel 96 Agarose (Invitrogen). Unpurified PCR amplicons were sequenced in both directions using M13 tails as primers. The sequencing reactions followed standard protocols of the Canadian Center for DNA Barcoding (Hajibabaei et al. 2005), with products subsequently purified using Agencourt CleanSEQ protocol (Agencourt) and processed using BigDye version 3.1 on an ABI 3730 DNA Analyzer (Applied Biosystems). Sequences were assembled with Sequencer 4.5 (GeneCode Corporation, Ann Arbor, MI, USA) and aligned by eye using BIOEDIT version 7.0.5.3 (Hall 1999). We observed no indels in this coding region of the mitochondrial genome and therefore all base positions were aligned with confidence in positional homology. Distance analyses were conducted with MEGA4 (Tamura et al. 2007) using a neighbor-joining (Saitou and Nei 1987) algorithm and distances corrected with the Kimura-2 parameter (Kimura 1980). The robustness of nodes was evaluated through bootstrap analysis of 1000 pseudoreplicates.

### Cybertaxonomy.

The present paper demonstrates several innovative methods of semantic tagging and semantic enhancements, text and data processing, publishing and dissemination in taxonomy, described in more detail in a forum paper published in the same issue (Penev et al. 2010). Among the most important semantic enhancements shown in the paper are: fine granularity XML (eXtensible Markup Language) tagging based on the US National Library of Medicine’s DTD (Document Type Definitions) TaxPub extension (the tagging of the present paper was based on TaxPub Version 123, http://sourceforge.net/projects/taxpub); final XML output of the paper validated against the NLM DTD TaxPub for archival in PubMedCentral and dissemination in XML to various aggregators, e.g., new descriptions to Encyclopedia of Life (http://www.eol.org) and all taxon treatments to Plazi (http://www.plazi.org); vizualisation of main tag elements within the text (e.g., taxon names, taxon treatments, DNA sequences, localities, type materials, etc.); mapping of localities listed in the whole paper or within separate taxon treatments; a dynamically created Pensoft Taxon Profile (PTP) page for each taxon name mentioned in the paper; Genbank accession numbers autotagged and linked to the National Center for Biotechnology Information (NCBI, http://www.ncbi.nlm.nih.gov) and Barcode of Life (BOLD, http://www.boldsystems.org); external links of references to PubMed, Google Scholar, Biodiversity Heritage Library and/or other sources.

All 70 images included in this publication have been deposited in MorphBank (Appendix D doi:10.3897/zookeys.50.504-app.D). All the revised species were registered in ZooBank and Life Science Identifiers (LSID) were assigned to them. Accession numbers were obtained from BOLD (see Appendix C doi:10.3897/zookeys.50.504-app.C for complete metadata) and GenBank for all COI gene sequences. Datasets in spreadsheet format for specimen localities have been shared with the Global Biodiversity Information Facility (GBIF, http://www.gbif.org) via Appendix A doi:10.3897/zookeys.50.504-app.A. To illustrate all records of the species in North Africa interactively in Google Earth, KML files were generated and are available for download as Appendix E doi:10.3897/zookeys.50.504-app.E. The interactive key for identification of all currently valid species of Eupolybothrus was made with DELTA software http://delta-intkey.com (Appendix F doi:10.3897/zookeys.50.504-app.F).

### Abbreviations:

ODoriginal description; RDredescription; Kkey; FRfaunistic record; CHLchecklist or catalogue; BDbiological data.

T/TT – Tergite/Tergites, CCoxa, TrTrochanter, PFePrefemur, FeFemur, TiTibia; Letters a, m, p stand for spines in anterior, medial and posterior positions, respectively; those in brackets indicate the variable spines.

LI, II, III, IV stand for larval stadia 1, 2, 3, 4, respectively. PLI, II, III, etc. stand for post-larval stadia 1, 2, 3, etc. subad. = subadult; juv. – juvenile.

## Results

### Taxonomic account of the currently valid African species of Eupolybothrus

Order Lithobiomorpha Pocock, 1895

Family Lithobiidae Newport, 1844

Subfamily Ethopolyinae Chamberlin, 1915

#### Genus Eupolybothrus

Verhoeff, 1907

urn:lsid:zoobank.org:act:9A79187E-FC75-4CB7-B634-ABBD4B471D2A

##### Type species.

Lithobius grossipes C.L. Koch, 1847, by subsequent designation of Chamberlin (1925). Type locality: Triest, Italy.

##### Diagnosis.

Medium- to large-sized Lithobiidae (body length 16–48 mm) with numerous irregularly arranged pores on the last four pairs of coxae; antennal articles always more than 20, from 38 to around 80; ocelli numerous, usually from 1+16 to 1+24, absent or reduced in some troglobitic species; porodont situated laterad to the forcipular coxosternal teeth; tergites with or without posterior triangular projections, tarsi of all legs bipartite; forcipular coxosternite with 5+5–14+14 teeth (usually from 7+7 to 10+10); female gonopod with 2 spurs and simple claw, male gonopods single or bipartite.

##### Assigned valid species (20).

Eupolybothrus (Eupolybothrus) andreevi Matic, 1964, Eupolybothrus (Schizopolybothrus) caesar (Verhoeff, 1899), Eupolybothrus (Propolybothrus) dolops Zapparoli, 1998, Eupolybothrus (Schizopolybothrus) excellens (Silvestri, 1894), Eupolybothrus (Eupolybothrus) fasciatus (Newport, 1845), Eupolybothrus (Eupolybothrus) gloriastygis (Absolon, 1916), Eupolybothrus (Eupolybothrus) grossipes (C.L. Koch, 1847), Eupolybothrus kahfi Stoev & Akkari sp. n., Eupolybothrus (Parapolybothrus) herzegowinensis (Verhoeff, 1900), Eupolybothrus (Parapolybothrus) imperialis (Meinert, 1872), Eupolybothrus (Schizopolybothrus) leostygis (Verhoeff, 1899), Eupolybothrus (Eupolybothrus) litoralis (L. Koch, 1867), Eupolybothrus (Eupolybothrus) longicornis (Risso, 1826), Eupolybothrus (Allopolybothrus) nudicornis (Gervais, 1837), Eupolybothrus (Parapolybothrus) obrovensis (Verhoeff, 1930), Eupolybothrus (Mesobothrus) transsylvanicus (Latzel, 1882), Eupolybothrus (Schizopolybothrus) tabularum Verhoeff, 1937, Eupolybothrus (Leptopolybothrus) tridentinus (Fanzago, 1874), Eupolybothrus (Propolybothrus) werneri (Attems, 1902), Eupolybothrus (Mesobothrus) zeus (Verhoeff, 1901).

##### Taxa of uncertain taxonomic status (10).

Eupolybothrus (Schizopolybothrus) acherontis (Verhoeff, 1900), Eupolybothrus acherontis wardaranus (Verhoeff, 1937), Eupolybothrus (Mesobothrus) macedonicus (Verhoeff, 1943), Eupolybothrus osellai Matic, Floca & Hurezeanu, 1992, Eupolybothrus ruffoi Matic, Floca & Hurezeanu, 1992, Eupolybothrus sketi Matic, 1979, Eupolybothrus (Schizopolybothrus) spiniger (Latzel, 1888), Eupolybothrus (Schizopolybothrus) stygis (Folkmanova, 1940), Eupolybothrus valkanovi (Kaczmarek, 1973), Eupolybothrus (Propolybothrus) verrucosus (Sseliwanoff, 1876).

##### Remarks.

Several taxa assigned to Eupolybothrus remain species inquirendae. Here we briefly review the current status of these taxa. Eupolybothrus stygis was described from Iljina pećina (cave) near Trebinje in Bosnia and Herzegovina (Folkmanova 1940), and Stoev (2001b) suggested that it could be identical with Eupolybothrus leostygis, a troglobitic species known from the same area (Eason 1983). In the key below it keys out together with Eupolybothrus acherontis, another poorly known species from Bosnia and Herzegovina. Stoev (2001a,b) noted that Eupolybothrus spiniger, Eupolybothrus acherontis and Eupolybothrus acherontis wardaranus could be identical with Eupolybothrus caesar. Being the oldest available name, in case of synonymy Eupolybothrus spiniger would have priority over Eupolybothrus caesar. Thanks to Verena Stagl, curator of myriapods at NHMW, we were able to obtain a photograph of the prefemur of the ultimate leg pair of male Eupolybothrus spiniger which shows no differences with that of Eupolybothrus caesar. However, until we personally examine the types we prefer to treat Eupolybothrus spiniger and Eupolybothrus caesar as separate species. Eupolybothrus valkanovi was based on a single female with unusually short gonopodial spurs found near Asenovgrad, Rhodope Mts in Bulgaria (Kaczmarek 1973). According to Stoev (2002) it is most likely conspecific with the morphologically similar Eupolybothrus transsylvanicus which is also known to occur in the area. Eupolybothrus sketi was described from male and female specimens found in the Jakupica Mts, in the Former Yugoslav Republic of Macedonia (Matic 1979). It is listed under the possible synonyms of Eupolybothrus transsylvanicus by Minelli (2006) but its status is yet to be clarified. Morphologically, it is most closely related to Eupolybothrus zeus from Greece. Eupolybothrus tabularum was synonymised under Eupolybothrus excellens by Minelli and Zapparoli (1985) but was recently found to be a good species. A paper on this subject is currently in preparation by M. Zapparoli and will be published elsewhere. Although both species share some traits in common (like 15VCm spine), the long median protuberance on the prefemur of leg 15 in males convincingly distinguishes Eupolybothrus excellens from Eupolybothrus tabularum (see key below). Eupolybothrus macedonicus is hitherto known only from its type locality, Temna cave near Loutraki, North Greece (Zapparoli 2002). Likewise, Eupolybothrus verrucosus is presently known only from its original description based on a single female specimen from Moldova (Minelli 2006). The taxonomic status of both species remains uncertain. The status of Eupolybothrus cloudsley-thompsoni, Eupolybothrus osellai and Eupolybothrus ruffoi is addressed under Discussion.

##### Eupolybothrus nudicornis

(Gervais, 1837)

urn:lsid:zoobank.org:act:3C17C879-5D17-470E-BD13-C07E14F44534

[Fig F1] Figs 1–2

Lithobius nudicornis Gervais 1837: 49. Type locality: Sicily, Italy. **OD**.
Lithobius impressus CL Koch 1841: 224, Tab. IX. Type localities: Alger and Oran, Algeria. **OD**.
Lithobius impressus : Lucas 1849: 340, Pl. 2, fig. 4.**RD**, **FR**.
Lithobius monilicornis Newport in Lucas 1849: 384. Type locality: Boudjaréa, near Algiers, Algeria. **OD**.
Lithobius elongatus Newport in Lucas 1849: 385. Type localities: Lac Tonga, Lac Houbeira, La Calle, all in Algeria. **OD**.
Lithobius impressus : L Koch 1862: 36, Tab. I, Fig. 7 a, b. **RD**.
Lithobius impressus : CL Koch 1863: 115, Tab. LII, Fig. 105. **RD**.
Lithobius impressus : Meinert 1872: 308. **FR**, **BD**.
Lithobius koenigi : Verhoeff 1891: 65. Type locality: Tunis. **OD**.
Lithobius impressus : Pocock 1892: 25. **FR**.
Lithobius Hemilithobius elongatus : Silvestri 1896: 148. **RD**, **FR**, **BD**.
Lithobius elongatus : Verhoeff 1901: 438. Type localities: Algeria: Oran: Rio Salado, Chaba el Ham, Djebel el Tessala, Saida, Hamman bou Hadjar. **OD**.
Polybothrus Koenigi Verh.: Attems 1908: 104. **FR**.
Bothropolys impressus , ? Bothropolys monilicornis, ? Lithobius elongatus, ? Lithobius nudicornis : Brolemann 1921: 105. **CHL**.
Bothropolys elongatus : Brolemann 1931a: 131. **FR**.
Bothropolys elongatus , Bothropolys elongatus : Brolemann 1932: 54. **K**.
Bothropolys elongatus Königi : Manfredi 1939: 110. **FR**.
Eupolybothrus Allopolybothrus cloudsley-thompsoni Turk 1955: 281, figs 7–12. Type locality: Roman aqueduct, 15 miles south of Tunis, Tunisia. **OD**. New Synonym!
Eupolybothrus cloudsley-thompsoni : Cloudsley-Thompson 1956: 327, Appendix VIII. **CHL**.
Schizopolybothrus lebruni Dobroruka 1968: 213, fig. 17. Type locality: Reg. Pont du Fahs, Djebel Mansour. **OD**.
Eupolybothrus impressus : Eason 1972a: 108. **RD**.
Eupolybothrus impressus , Eupolybothrus impressus : Eason 1972b: 305–306. **RD**.
Eupolybothrus impressus : Zapparoli 1985: 1, figs 1–2. **FR**, **RD**.
Bothropolys elongatus : Daas et al. 1995: 21, 23, 24, 25, 26. **FR**, **BD**.
Eupolybothrus elongatus : Daas et al. 1996: 365–370. **FR**, **BD**.
Eupolybothrus nudicornis : Daas et al. 2003: 240. **BD**.
Lithobius koenigi : Stagl and Zapparoli 2006: 19. **CHL**.

###### Material examined.

Type material of Eupolybothrus cloudsley-thompsoni: 7 ♂♂, 2 ♀♀, 1 juv. of Eupolybothrus cloudsley-thompsoni + 1 juv. Lithobius castaneus Newport, 1844, Tunisia, Roman aqueduct 15 miles south of Tunis, 4.IV.1954, Cloudsley-Thompson leg. Turk Collection, Syntypes. Last pair of legs mounted on a slide, Turk collection 1984.10.1.77 (NHM).

Nontype material: SPAIN: 3 ♂♂, 3 ♀♀, 5 subad. ♂♂, 3 subad. ♀♀, 2 juv., labelled “ Lithobius impressus Granada Meinert” and “ Eupolybothrus impressus (Newport) det. E.H. Eason 1980”, Meinert Collection (ZMUC); ALGERIA: 3 specimens, Djebel Maadid, Kalas Beni Hammaad, 1000 m, 23.X.1989, G. Osella (CMZ); TUNISIA (governorates listed according to their location, from North to South): Bizerte Governorate: 8 ♂♂, 7 ♀♀, 2 juv., Ghar el Melh, garden, Ceratonia siliqua, 37°19'N, 09°51'E, alt. 35 m, in litter, 11.I.2003, N. Akkari leg. (FMNH); 1 ♂, 2 subad. ♀♀, La Grotte beach, 37°19'N, 09°50'E, alt. 5 m, slope facing the sea, halophilous vegetation, under stones, 12.II.2004, N. Akkari leg. (FMNH); 4 ♂♂, 2 ♀♀, Ghar el Melh, 37°19'N, 09°51'E, alt. 35 m, slope with sparse shrubs, under stones, 1.III.2004, N. Akkari leg. (FMNH); 1 ♂, 1 ♀, Ichkeul National Park, inside the park, 37°07.861'N, 09°41.338'E, alt. 5 m, rocks, shrubs, grass, close to the road, under stones, 23.III.2008, P. Stoev, N. Akkari leg. (NMNHS); 1 ♂, 1 ♀, Ichkeul National Park, 37°08.25'N, 09°41.31'E, alt. 0–50 m, Olea europaea- Pistacia lentiscus maquis, 12.III.2009, N. Akkari, H. Enghoff leg. (ZMUC); Béja Governorate: 3 ♂♂, 8 ♀♀, 3 subad. ♂♂, Rahayette (2 km from Sidi Salem Lac), 36°42'N, 09°18'E, alt. 180 m, meadow, shrubs, under stones, 26.XII.2003, N. Akkari leg. (FMNH); 1 ♂, 3 juv., entrance of the city, 36°42.7311'N, 09°19.611'E, alt. 362 m, open area with scattered Eucalyptus, under stones, 24.IV.2005, N. Akkari leg. (FMNH); 5 ♂♂, 7 ♀♀, 7 km from Zahret Médine, 36°46.857'N, 09°01.688'E, alt. 500 m, limestone hill, shrubs, under stones, 20.III.2008, P. Stoev, N. Akkari leg. (NMNHS); 1 ♀, entrance of Béja City, 36°42.311'N, 09°19.611'E, alt. 362 m, open area with scattered Eucalyptus, under stones, 15.IV.2007, N. Akkari leg. (FMNH); Tunis Governorate: 4 ♂♂, 2 juv.,Jebel Bou Kornine, close to the asphalt road (highway: Tunis-Hammamète) 17.II.2004, N. Akkari leg. (FMNH); 1 juv., Tunis, 12.II.1903, Lajos Biró leg (HNHM); 1 ♂, 1 subad. ♂, Tunis, 23.II.1903, Lajos Biró leg. (HNHM); 4 ♂♂, 2 ♀♀, Jebel Bou Kornine, 8.IV.2004, N. Akkari leg. (FMNH); 4 ♂♂, 4 ♀♀, Jebel Bou Kornine, 8.IV.2007, N. Akkari leg. (FMNH); 1 ♀, Bou Kornine National Park, 36°42.530'N, 10°20.680'E, alt. 105–150 m, Thuja, Eucalyptus, dry river bed, under stones and logs, 4.III.2008, P. Stoev, N. Akkari leg (NMNHS); Ariana Governorate: 1 ♂, 2 ♀♀, Nahli Park, 36°53'N, 10°09'E, alt. 68 m, suburban habitat with Eucalyptus, Pinus halepensis, under stones, 31.III.2006, N. Akkari leg. (FMNH); 1 ♂, 1 ♀, Sidi Thabet, Jebel Ammar, 10.II.2007, N. Akkari leg. (FMNH); Jendouba Governorate: 1 ♂, Béni Mtir, surroundings of the dam, 36°44'N, 08°44'E, alt. 445 m, shrubs, under stones, 18.II.2007, N. Akkari leg. (FMNH); 3 ♂♂, 3 ♀♀, Béni Mtir, 36°44'N, 08°44'E, alt. 408 m, slope close to the asphalt road, Quercus suber, under stones, 19.II.2007, N. Akkari leg. (FMNH); 3 ♀♀, same locality, under logs, 36°44.583'N, 08°44.832'E, alt. 493 m, 19.II.2007, N. Akkari leg. (FMNH); 5 ♀♀, same locality, 36°44.006'N, 08°44.001'E, alt. 500 m, 19.II.2007, N. Akkari leg. (FMNH); 4 ♂♂, 4 ♀♀, same locality, alt. 503 m, Quercus suber, Erica arborea and Myrtus communis, under stones, 19.II.2007, N. Akkari leg. (FMNH); 3 subad. ♂♂, Béni Mtir and surroundings, 36°43.888'N, 08°44.105'E, alt. 404 m, Quercus suber, close to the road, under stones, 21.III.2008, P. Stoev, N. Akkari leg. (BIO); 4 ♂♂, 3 ♀♀, 2 subad. ♂♂, ruins of the ancient Roman town Bulla Regia, 36°33.506'N, 08°45.356'E, alt. 185 m, under stones, 21.III.2008, P. Stoev, N. Akkari leg. (NMNHS); 2 subad. ♂♂, 1 subad. ♀, Tabarka, the Genoese fort and surroundings, 36°57.838'N, 08°44.680'E, alt. 20–30 m, slope facing the sea, grass, rocks scattered trees, under stones and logs, 22.III.2008, P. Stoev, N. Akkari leg. (NMNHS); 2 ♂♂, 2 ♀♀, Hammam Bourguiba (west of Aïn Draham), 36°45.926'N, 08°35.084'E, alt. 158 m, meadow with scattered trees, under stones, 22.III.2008, P. Stoev, N. Akkari leg. (NMNHS); 1 ♀, 3 km from Hammam Bourguiba (west of Aïn Draham), 36°46.476'N, 08°36.575'E, alt. 322 m, meadow with scattered trees, under stones, 22.III.2008, N. Akkari, P. Stoev leg. (NMNHS); 1 ♂, 2 subad. ♂♂, 3 subad. ♀♀, 9 km from Hammam Bourguiba (west of Aïn Draham), 36°48.046'N, 08°39.544'E, alt. 379 m, pine forest, humid, close to river, under stones, logs and leaf litter, 22.III.2008, P. Stoev, N. Akkari leg. (NMNHS); 2 ♀♀, surroundings of Aïn Draham, collected from under bark of decaying trunk and stones, 31.III.1977, L. Gozmány, S. Mahunka leg. (HNHM); 3 ♂♂, 3 ♀♀, Tabarka, 36°58.105'N, 08°45.356'E, alt. < 40 m, coastal slope below Genoese fort, under stones, 9.III.2009, N. Akkari, H. Enghoff leg. (ZMUC); Nabeul Governorate: 1 ♂, 1 ♀, 1 juv., Cap Bon Peninsula, Hammamète, Olea europaea orchard, under stones, 17.II.2004, N. Akkari leg. (FMNH); 2 ♂♂, 2 ♀♀, Cap Bon Peninsula, Dar Chichou, coniferous forest, under stones with Pinus halepensis, 14.III.2004, N. Akkari leg. (FMNH); 1 ♂, 4 ♀♀, 1 juv., Cap Bon Peninsula, El Haouaria, N37°3, E10°59, alt. 50 m, 28.IV.2004, N. Akkari leg. (FMNH); 1 ♂, 3 ♀♀, 3 juv., Cap Bon Peninsula, Oued el Abid, close to the sea shore, under stones, 9.III.2005, N. Akkari leg. (FMNH); 3 ♂♂, 3 juv., Cap Bon Peninsula, Oued el Abid village, 36°51.804'N, 10°44.701'E, alt. 79 m, 9.III.2005, N. Akkari leg. (FMNH); 1 ♂, Cap Bon Peninsula, Sidi Erraiès, close to a polluted beach, 9.III.2005, N. Akkari leg. (FMNH); 1 ♂, 2 ♀♀, Cap Bon Peninsula, Kélibia, Mansoura beach, 36°50.994'N, 11°07.361'E, alt. 1 m, under stones, 23.III.2005, N. Akkari leg. (FMNH); 3 ♂♂, 1 ♀, 1 subad. ♂, 2 subad. ♀♀, Cap Bon Peninsula, near Oued El Abid Dam, 36°49.901'N, 10°42.378'E, alt. 42 m, grass, stones, under stones, 24.III.2008, N. Akkari, P. Stoev leg. (NMNHS); 12 ♂♂, 10 ♀♀, 1 subad. ♂, 2 juv., Cap Bon Peninsula, near Oued El Abid village, 36°51.804'N, 10°44.711'E, alt. 79 m, Eucalyptus and Pinus forest, under stones, 24.III.2008, P. Stoev, N. Akkari leg. (NMNHS); 1 ♂, Cap Bon Peninsula, 20 km from El Haouaria, 36°56.660'N, 10°53.321'E, alt. 30 m, broad leaf forest, sandy soil, under stones, 24.III.2008, N. Akkari, P. Stoev leg. (BIO); 8 ♂♂, 11 ♀♀, 1 subad. ♀, El Haouaria, the ancient Roman quarry and surroundings, 37°03.448'N, 10°59.869'E, alt. 51 m, slope facing the sea, under stones, 24.III.2008, P. Stoev, N. Akkari leg. (NMNHS); 1 ♂, 1 ♀, Cap Bon Peninsula, El Haouaria, coast, 37°03.448'N, 10°59.869'E, alt. 2 m, rocks, sand, 10–50 m from the water line, under stones, 25.III.2008, N. Akkari, P. Stoev leg. (NMNHS); 2 ♀♀, Cap Bon Peninsula, Kélibia, plage El Mansoura, 36°51.046'N, 11°07.343'E, alt. 5–10 m, approx. 100 m from the water line, Oleander, Mimosa, under stones and in leaf litter, 25.III.2008, P. Stoev, N. Akkari leg. (BIO); 1 ♀, Cap Bon Peninsula, Kélibia, the fort and surroundings, 36°50.337'N, 11°06.841'E, alt. 10–40 m, slope, Eucalyptus, Mimosa, shrubs, under stones, 25.III.2008, N. Akkari, P. Stoev leg. (NMNHS); 2 ♂♂, 2 ♀♀, Cap Bon Peninsula, 7 km from Menzel Bou Zelfa, 36°40.268'N, 10°40.677'E, alt. 236 m, Pinus, Quercus, shrubs, under stones, 25.III.2008, P. Stoev, N. Akkari leg. (BIO, NMNHS); 8 ♂♂, 2 ♀♀, Cap Bon Peninsula, El Haouaria, Roman cave, 1.IV.2007, K. Tajovský leg. (FMNH); 1 juv., Cap Bon Peninsula, Jebel Abderrahman, Olea europaea orchard, under stones, 12.XI.2006, N. Akkari leg. (FMNH); Zaghouan Governorate: 3 ♂♂, 2 ♀♀, Jebel el Fahs, 36°22.39'N, 09°53.41'E, alt. 172 m, under stones, 20.III.2006, N. Akkari leg. (FMNH); 1 ♀, Jebel el Fahs, 36°24.298'N, 10°09.057'E, alt. 166 m, suburbs, Olea europaea orchard, under stones, 25.II.2007, N. Akkari leg. (FMNH); 1 ♀, Jebel Zaghouan, under stones, 17.III.2007, N. Akkari leg. (FMNH); 1 ♂, 1 ♀, 2 subad. ♂, 1 subad. ♀, Jebel Zaghouan, surroundings of the marabout Sidi Bou Gabrine, 36°22.423'N, 10°06.328'E, alt. 642 m, meadows, scattered trees, under stones and in leaf litter, 17.III.2008, N. Akkari leg. (1♂, 1♀ – BIO; 2 subad. ♂♂, 1 subad. ♀ – NMNHS); 1 ♀, Jebel Zaghouan, surroundings of the marabout Sidi Bou Gabrine, 36°22.423'N, 10°06.328'E, alt. 642 m, meadows, scattered trees, under stones, 29.III.2008, P. Stoev, N. Akkari leg. (NMNHS); 1 ♀, Jebel Zaghouan, surroundings of the Gouffre du courant d’air (small limestone cave), 36°21.980'N, 10°05.513'E, alt. 561 m, Quercus ilex, Pistacia lentiscus, Jasminum fructicans, under stones and in leaf litter, 17.III.2008, N. Akkari, P. Stoev leg. (NMNHS); 5 ♂♂, 4 ♀♀, Jebel Zaghouan, collecting along the track between Gouffre Anti Prehistorique (36°21.595'N, 10°05.208'E) and Sidi Bou Gabrine (36°22.423'N, 10°06.328'E), 500–700 m, mixed forest, under stones and in leaf litter, 18.III.2008, N. Akkari, P. Stoev leg. (NMNHS); 2 ♂♂, Jebel Zaghouan, collecting along the track Sidi Bou Gabrine (36°22.423'N, 10°06.328'E) – Sidi Abdel kader Cave (36°22.419'N, 10°06.371'E) – Saida Mannoubia (36°22.650'N, 10°06.332'E) – the asphalt road to Zaghouan (36°22.924'N, 10°06.789'E), alt. 650–780 m, mixed forest, under stones and in leaf litter, 19.III.2008, P. Stoev, N. Akkari leg. (NMNHS); Le Kef Governorate: 4 ♂♂, 2 ♀♀, 1 juv., Tajerouine, Bou Yagoum dam, 35°53'N, 08°53'E, alt. 650–700 m, open dry habitat, under stones, 16.III.2005, N. Akkari leg. (FMNH); 1 ♂, 2 ♀♀, Ferme Shitta, Jebel Eddyr, about 6 km NNE from Le Kef, alt. about 1100 m, collected from sward, mosses, from below stones imbedded in grassy soil, in shaded sites between cliff walls, 28.III.1977, L. Gozmány, S. Mahunka leg. (HNHM); 3 ♂♂, 1 subad. ♂, 1 subad. ♀, 2 juv., Ferme Shitta, Jebel Eddyr, about 7 km NNE from Le Kef, alt. about 1100 m, collected from under rocks at feet of cliff walls, 26.III.1977, L. Gozmány, S. Mahunka leg. (HNHM); 3 ♂♂, 1 ♀, 1 subad. ♂, 1 subad. ♀, Nebeur, about 30 km N from Le Kef, collected on ground and under stones on steep banks and in riverbed, 30.III.1977, L. Gozmány, S. Mahunka leg. (HNHM); 2 subad. ♂♂, 2 juv., 1 larva, Dugga Archeological site, under stones, 30.X.2009, N. Akkari leg. (ZMUC); 1 ♂, South of Le Kef, a flat, immense, fungiform limestone hill, collected from crags, 27.III.1977, L. Gozmány, S. Mahunka leg. (HNHM); Siliana Governorate: 17 ♂♂, 7 ♀♀, 4 subad. ♀♀, Jebel Bargou, 5 km from Bargou (road Bargou – Ouslatia), 36°05.775'N, 09°37.347'E, alt. 571 m, Quercus, Olea, shrubs, under stones, 28.III.2008, N. Akkari, P. Stoev leg. (2 ♂♂, 2 ♀♀ – BIO; remaining in NMNHS); 4 ♂♂, 9 ♀♀, 1 subad. ♀, Jebel Bargou,50 km from Ouslatia (road Bargou – Ouslatia), 36°06.941'N, 09°39.392'E, alt. 512 m, sparse olive trees, rocks, under stones, 28.III.2008, P. Stoev, N. Akkari leg. (NMNHS); 1 ♂, Kesra, 36°51'N, 09°12'E, alt. 850–900 m, 12.V.2005, coniferous forest, under stones, N. Akkari leg. (FMNH); Kairouan Governorate: 1 subad. ♂, Sbikha village, under stones, 10.XII.2006, N. Akkari leg. (FMNH); 1 ♂, 3 ♀♀, same locality, under stones, 2.II.2007, N. Akkari leg. (FMNH); 1 ♂, 2 subad. ♀♀, 1 juv., 2 larvae, 6 km from Ouslatia, 35°51.785'N, 09°30.972'E, alt. 581 m, sparse olive trees, Roman ruins, bush, open area, stone debris, under stones, 6.III.2008, N. Akkari, P. Stoev leg. (NMNHS); 3 ♂♂, 1 ♀, Thuburbo Majus, open area with shrubs, 30.III.2007, K. Tajovský leg. (FMNH); Sousse Governorate: 3 ♂♂, Sidi Khalifa (67 km from Tunis), open area with scattered shrubs, 17.II.2004, N. Akkari leg. (NMNHS); 2 ♂♂, 2 ♀♀, 2 subad. ♀♀, Hergla, 35°59.735'N, 10°26.300'E, close to the asphalt road, under stones, 22.III.2005, N. Akkari leg. (FMNH); 2 ♂♂, Bou Ficha, Ken, 36°15.511'N, 10°26.617'E, alt. 15 m, close to a saline depression, under stones, 22.III.2005, N. Akkari leg. (FMNH); Monastir Governorate: 3 subad. ♂♂, 3 subad. ♀♀, Békalta, 35°37'N, 11°00'E, alt. 16 m, sandy soil close to the asphalt road, under stones, 30.XI.2003, N. Akkari leg. (FMNH); Kasserine Governorate: 1 juv., Sbeitla, 30 km NW from Kasserine, inside the ruins of the ancient Roman town of Sifetoula, under stones, 7.III.2008, P. Stoev, N. Akkari leg. (NMNHS); 2 ♂♂, 1 ♀, Chambi National Park, surroundings of the park’s guest house, 35°10.139'N, 08°40.486'E, alt. 950 m, sparse trees, bush, Pinus halepensis, under stones, 7.III.2008, N. Akkari, P. Stoev leg. (NMNHS); 1 ♀, 1 subad. ♂, Chambi National Park, surroundings of the park’s guest house, 35°10.139'N, 08°40.486'E, alt. 950–1000 m, Pinus halepensis, Stipa tenacissima, Thuja, under stones, logs and leaf litter of Pinus halepensis, 8.III.2008, P. Stoev, N. Akkari leg. (NMNHS); 1 ♂, Chambi National Park, inside the park, 35°11.901'N, 08°39.505'E, alt. 1291 m, Pinus halepensis, Quercus ilex, Stipa tenacissima, slope, under stones and in leaf litter, 9.III.2008, P. Stoev, N. Akkari leg. (NMNHS); 1 ♂, 1 ♀, juv., Chambi National Park, Chambi peak and its surroundings, 35°12.285'N, 08°40.653'E, alt. 1500–1540 m, rocks, sparse Pinus trees, under stones, 8.III.2008, N. Akkari, P. Stoev leg. (NMNHS); 2 ♀♀, 5 subad. ♂♂, 1 subad. ♀, 1 juv.,Chambi National Park, inside the park, 35°11.935'N, 08°40.418'E, alt. 1468 m, Pinus halepensis, Quercus ilex, Stipa tenacissima, slope, under stones and in leaf litter, 9.III.2008, N. Akkari, P. Stoev leg. (NMNHS); 3 ♂♂, 1 ♀, subad. ♂, ♀, 6 juv., Chambi National Park, Chambi peak and its surroundings, 35°12.285'N, 08°40.653'E, alt. 1500–1540 m, Pinus halepensis, Quercus ilex, Stipa tenacissima, under stones and in leaf litter, 9.III.2008, P. Stoev, N. Akkari leg. (2 ♂♂, 2 juv. – BIO; 1 ♂, 1 ♀, subad. ♂, ♀, 4 juv. – NMNHS); Mahdia Governorate: 8 ♂♂, 9 ♀♀, 1 subad. ♀, 1 larva, Mahdia, touristic area, 35°32.796'N, 11°01.662'E, alt. 0 m, scattered palm trees and shrubs close to the road, polluted area not far from agricultural land, under stones, 16.III.2008, N. Akkari, P. Stoev leg. (NMNHS); 2 ♂♂, 2 ♀♀, 1 juv., surroundings of Ksour Essef (17 km from Mahdia), 35°24.824'N, 10°58.026'E, alt. 59 m, olive trees (Olea europaea), grass, stones and shrubs, under stones, 16.III.2008, N. Akkari, P. Stoev leg. (NMNHS); Sfax Governorate: 2 ♂♂, 1 ♀, Sfax, house’s garden, 23.III.2004, N. Akkari leg. (FMNH); Gafsa Governorate: 1 juv., 1 larva, Jebel Bou Ramli, 34°30.877'N, 08°39.731'E, alt. 512 m, deserted rocky plain at the foot of the mountain, scattered trees, Opuntia and palm trees, under stones, 10.III.2008, P. Stoev, N. Akkari leg. (NMNHS); Tozeur Governorate: 1 juv., Midès, 34°24.419'N, 07°54.896'E, alt. 376 m, dry rocky slope, under stones, 10.III.2008, N. Akkari, P. Stoev leg. (NMNHS); Gabès Governorate: 2 juv., Matmata, 33°32.450'N, 09°59.055'E, alt. 384 m, arid biotope, shrubs and stones, under stones, 13.III.2008, P. Stoev, N. Akkari leg. (NMNHS); Tataouine Governorate: 2 juv., surroundings of Tataouine City, 32°55.506'N, 10°26.913'E, alt. 293 m, arid biotope, slope, stones, scattered trees of Pinus (planted), under stones, 13.III.2008, P. Stoev, N. Akkari leg. (NMNHS); 1 larva? (damaged), Ksar Ouled Soltane, 32°47.281'N, 10°30.784'E, alt. 453 m, arid biotope, rocks, stones, close to the village, under stones, 14.III.2008, N. Akkari, P. Stoev leg. (NMNHS).

###### Description.

**Figure 1. F1:**
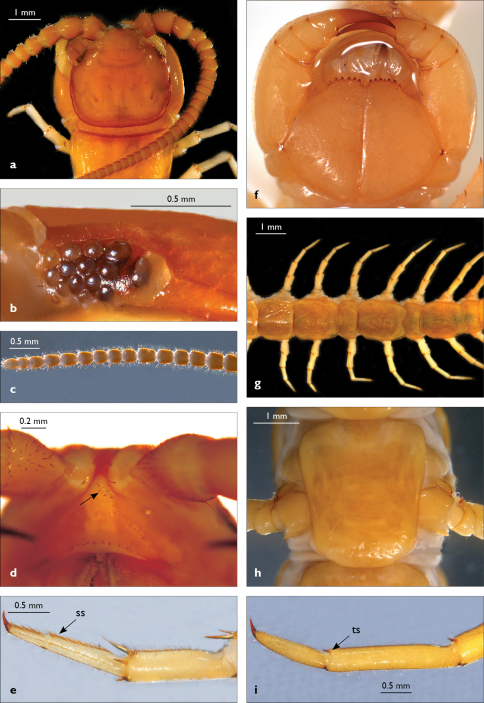
Eupolybothrus nudicornis: a – cephalic plate; b – ocelli and Tömösváry’s organ; c – apical part of antenna; d – clypeus; e – leg 10; f – forcipule; g – TT 6-13; h – sternite 7; i – tarsus 1 and tarsus 2 of leg 15, female from Chambi N.P. Clypeal setae indicated by an arrow (Fig. 1d). Fig. 1f without scale. ss – serial setae; ts – tarsal spine.

Length (from anterior margin of cephalic plate to posterior margin of telson) approx. 48 mm in largest specimens; cephalic plate slightly broader than long (Fig. 1a); head up to 2.7 mm long and up to 2.9 mm wide; leg 15 approx. 11.0 mm long, or approx. 27% length of body. Colour (after one and a half years in alcohol) uniformly light chestnut brown; legs and sternites yellow; only forcipular coxosternal teeth and posterior half of forcipular tarsungulum brown.

Cephalic plate finely punctate, with scattered minute setae, emerging from larger pits that form the punctae; slightly narrower than T1 (Fig. 1a); a median notch contributing to biconvex anterior margin; marginal ridge with a distinct median thickening occupying approx. 10–15% breadth of plate; posterior margin straight; transverse suture situated at about 1/3 of anterior edge; posterior limbs of transverse suture visible, connecting basal antennal article with anterior part of ocellararea. Ocelli: 1+3,4,4,1–1+3,4,4,2 pale, oval to elliptical, in 4 rows. Posterior (major) ocellus elliptical, much larger than seriate ocelli and situated well apart from them. Tömösváry’s organ very small (approx. 1/3 the size of adjacent seriate ocellus), circular, situated on faint sclerotisation lying close to anteriormost ocellus of third ocellar row (Fig. 1b).

Antennae moderately long, approx. 15 mm, reaching midline of T6 when folded backwards; approx. 37% length of body, composed of 43–44 articles; first three articles enlarged, with second being the largest (Fig. 1a); antenna gradually tapering towards the end; articles 5–20 broader than long, ultimate article of antenna about same length as penultimate or slightly longer (Fig. 1c). Basal two articles less setose than the others, which are densely covered with trichoid setae.

Clypeus (Fig. 1d) with a cluster of six medium-sized setae situated asymmetrically at the left half of its apex; central clypeal area smooth, without setae, basal part of clypeus with a single row of setae, lateral clypeal margins with very few dispersed setae.

Forcipule (Fig. 1f): coxosternite subhexagonal, lateral margins feebly convex; anterior margin set off as a rim by furrow that is impressed behind all teeth; coxosternal teeth 5+5, almost equal-sized; median diastema shallow, U-shaped; intradental distance varying, generally increasing towards lateral teeth; porodont arising from a small node at lateral coxosternal margin, situated below the dental rim, and well laterad from lateralmost tooth; base of porodont as thick as adjacent tooth; coxosternite smooth, with one or two rows of setae in close proximity to dental rim; dorsal side of coxosternite with sparse minute setae, the apices of which are not visible from the ventral side. Distal part of tarsungulum about 3–3.5 times longer than proximal part, devoid of setae. Forcipular trochanteroprefemur, femur and tibia fringed with few minute setae (Fig. 1f).

Tergites wrinkled (Fig. 1g); TT 11, 13 with well-developed posterior triangular projections, T9 without triangular projections, posterior angles right-angled; posterior margination poorly developed on all tergites, almost indistinguishable on some; T1 subtrapeziform, slightly wider than cephalic plate, almost as wide as T3. Posterior margin of T1 gently concave, those of TT 3, 5, 8, 10, 12, 14 moderately concave; on intermediate tergite deeply concave; posterior angles of TT 1–6, 8, 10 rounded; those of TT 7, 12, 14 right-angled; tergal setae tiny, almost indistinguishable, in general concentrated on the edges of tergites.

Sternites 1–14 elongated, subtrapeziform, finely punctate, with very few sparse setae, posterior margin convex (Fig. 1h); sternite 15 subrectangular, smooth and more densely setose, especially at posterior margin.

**Figure 2.**
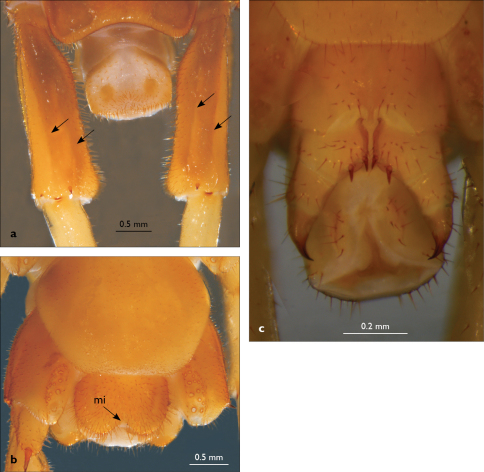
Eupolybothrus nudicornis: a – prefemora of leg-pair 15, dorsal view b – coxae and male first genital sternite c – female gonopods. Paramedian sulci indicated by arrows (Fig. 2a). mi – medial incision.

Legs: all legs moderately long (Fig. 1e); leg-pairs 13–15 longer than 1–12; leg 15 longest of all; maximal length of podomeres: coxa 1.2 mm, prefemur 2 mm, femur 1.8 mm, tibia 2.2 mm, tarsus 1 2.2 mm, tarsus 2 1.2 mm, pretarsus 0.3 mm. Tarsus 1 and tarsus 2 of legs 1–13 with two rows of ventral and two rows of lateral (on each side) serial setae (Fig. 1e); serial setae concentrated only on tarsus 2 of leg 14, absent on leg 15 (Fig. 1i). Pretarsus of legs 1–14 with a large principal claw and two smaller and thinner accessory ones emerging dorsally and basally from the principal claw; both accessory claws approx. 1/4–1/5 the length of principal claw, basal one generally smaller and thinner. Pretarsus of leg 15 with a large principal claw only (Fig. 1i). Leg 15 in males with secondary sexual modifications: prefemur moderately enlarged with two paramedian sulci not extending to distal end; distal part of prefemur swollen dorso-medially; inner side of prefemur more densely setose than the outer one (Fig. 2a). Male leg 14 with similar modifications but less pronounced. Spinulation: as in Table 1.

Coxal pores: small, circular, more concentrated on the outer part of pore-field, forming 3–4 irregular rows; only 2–3 pores from the internal row on each coxa larger; around 25–30 on legs 12–14 and about 17 pores on leg 15; pores of inner rows often separated by more than twice their own diameter, those of outermost row usually separated by less than their own diameter (Fig. 2b).

Male first genital sternite with emarginated posterior margin (Fig. 2b), posterior angles broadly rounded, sternal surface densely covered with numerous long brownish setae; gonopod hidden behind the edge of first genital sternite; small, basal part larger covered with six setae, apical part with 2 setae.

Female gonopods with 2+2 moderately long, apically pointed spurs and a simple, falcate claw (Fig. 2c). First article with approx. 14 setae concentrated on small protuberance at its posterior part; posterior half of second article with approx. 20 dorsal and dorso-lateral setae of various sizes; gonopodial claw with 5 moderately long lateral setae.

**Table 1:** Eupolybothrus nudicornis, Mahdia (Tunisia), adult male: spinulation of legs.

Leg	Ventral					Dorsal				
	C	Tr	PFe	Fe	Ti	C	Tr	PFe	Fe	Ti
1	-	-	mp	amp	amp	-	-	amp	a-p	a
2	-	-	mp	amp	amp	-	-	amp	a-p	a-p\
3	-	-	mp	amp	amp	-	-	amp	a-p	a-p\
4	-	-	mp	amp	amp	-	-	amp	a-p	a-p\
5	-	-	mp	amp	amp	-	-	amp	a-p	a-p\
6	-	-	mp	amp	amp	-	-	amp	a-p	a-p\
7	-	-	mp	amp	amp	-	-	amp	a-p	a-p\
8	-	-	mp	amp	amp	-	-	amp	a-p	a-p\
9	-	-	mp	amp	amp	-	-	amp	a-p	a-p\
10	-	-	amp	amp	amp	-	-	amp	a-p	a-p\
11	-	-	mp	amp	amp	-	-	amp	a-p	a-p\
12	-	m	amp	amp	amp	a	-	amp	(a)-p	a-p
13	-	m	amp	amp	amp	a	-	p	(a)-p
14	a	m	amp	amp	a-p	a	-	p	p
15	a	m	amp	am	-	a	-	(a)m	p	-

###### Post-embryonic development.

Meinert (1872) described immature stadia of Eupolybothrus nudicornis (sub Lithobius impressus), based on his study of specimens from Algeria, Granada (Spain) and Ischia (Italy). He recognized four classes:

- “Pullus” with 10 pairs of legs + 2 incompletely developed pairs (= LIII)

- “Pullus” with 12 pairs of legs + 3 incompletely developed pairs (= LIV)

- “Juvenis” (smaller PL)

- “Junior” (larger PL)

His information is summarized in Table 2. No information on the number of specimens in each group is available. Meinert’s data agree well with our own observations, except for the higher number of forcipular coxosternal teeth in the larval stadia.

**Table 2:** Character states in larval and postlarval stadia in Eupolybothrus nudicornis according to Meinert (1872, sub Lithobius impressus).

Stadium	Body length (mm)	Number of antennal articles	Number of ocelli	Number of forcipular coxosternal teeth
Pullus (LIII)	5-9	21	3	5+5 (also 4+5-4+4)
Pullus (LIV)		24-26	4	
Juvenis	9.5-11.8	25-29	4-6	4+5, 5+5
Junior	13.5-20	35-42	6-8	5+5, 6+6

Daas et al. (1996) studied post-embryonic development of Eupolybothrus nudicornis (sub Eupolybothrus elongatus Newport) in Algeria. The data from their table 2 are given here as Table 3. The first larval stadia were reared from eggs, whereas the older stadia were obtained from field-collected animals. By comparison to developmental series of other lithobiids, the numbers of legs given for LIII and LIV are anomalous (cf. Murakami 1958, Andersson 1981).

**Table 3:** Character states in larval and postlarval stadia in Eupolybothrus nudicornis according to Daas et al. (1996, sub Eupolybothrus elongatus). Each entry is based on at least four observations.

Stadium	Number of leg-pairs	Body length (mm)	Number of antennal articles
L0	7	5-6	9
LI	8	6.5	11-13
LII	9	7	15
LIII	11	7.5	17
LIV	13-14	8	21
PLI	15	9-11	34-36
PLII	15	13-15	38
PLIII	15	16-18	38-39
PLIV	15	19	39-40
PLV	15	21-23	40
PLVI	15	27-30	41-42
>PLVI	15	33-45	42-43

Further data on the post-embryonic development based on Tunisian specimens of Eupolybothrus nudicornis are provided by Silvestri (1896: 149, sub Lithobius elongatus) but they mostly refer to grown individuals and say nothing about the larval and earlier post-larval stadia (see Table 4).

In Table 4 we provide the character states for the different larval and postlarval stadia obtained from part of the studied material. The definition of postlarval stadia follows Daas et al. (1996) and is based on the length of respective specimen.

**Table 4:** Character states in larval and postlarval stadia in Eupolybothrus nudicornis in Tunisia according to Silvestri (1896) and new data. Silvestri’s data marked with an asterisk.

Stadium	Number of leg pairs	Body length (mm)	Number of antennal articles	Number of ocelli	Number of forcipular coxosternal teeth	Sex	Locality
LIV	12	7	26	3	4+4		Gafsa, Jebel Bou Ramli
	12	8	25	4	4+4		Archeological site of Dugga
	12	9	25	5	4+4		6 km from Ouslatia
PLI	15	8	30	4	4+4		Mides
PLII	15	10	30	5	5+5		6 km from Ouslatia
PLIII	15	14	41	7-8	5+5	FF	Chambi National Park
	15	15	38	6-7	5+5	MM	Jebel Bou Kornine
	15	16	37-38	7-8	5+5	MM	Archeological site of Dugga
	15	17	-	7	5+5	MM	Jebel Bou Kornine
	15	18	44-46	7-8	6+6	MM	Chambi National Park
PLIV	15	18	44-46	10-411	6+6	FF	Chambi National Park
		23	44	10	5+5	MM	Tabarka*
		23	40	8	5+5	FF	Tabrka*
		23	46	9	6+6	FF	Tabarka*
	15	24	40-46	11	5+6	FF	Beni Mtir
		25	44	10	6+6	FF	Tunis*
	15	26	45	11	5+5	MM	Ghar El Melh
		27	42	11	6+5	MM	Tunis*
		28	41	13	6+6	FF	Souk el Araba=Jendouba
		28	44	10	5+5	MM	Tunis*
PLV	15	28	41-44	12	5+5	MM	Dar Chichou
	15	25	41-42	14	6+6	MM	Chambi National Park
		29	44	12	5+5	MM	Souk el Arba=Jendouba*
		30	43	13	5+5	MM	Babouch*
PLVI	15	30	45-47	13-14	6+5	MM	Ghar El Melh
		35	44	10	6+5	MM	Souk el Arba=Jendouba*
	15	35	40	12-14	6+6	MM	Beni Mtir
	15	38	42-43	13-14	5+5	FF	Beja
		40	41	10	6+6	FF	Tunis*
	15	40	-	14-15	5+5	MM	Ghar El Melh
	15	42	-	11-13	5+5	MM	Jebel Bou Kornine
	15	43	44	13	6+6	MM	Sidi Khalifa
	15	48	40-43	11-13	7+7	MM	Sidi Khalifa

###### Distribution in Tunisia (Map 1).

Eupolybothrus nudicornis is widespread in the Humid, Subhumid, Semiarid and Arid bioclimatic zones, according to the bioclimatic division of Tunisia of Emberger (1966). The species occurs in the northwestern mountains of Kroumirie and Mogods (Aïn Draham, Béni Mtir, Hamman Bourguiba, Béja) where it interconnects with the populations in northern Algeria (La Calle, Constantine, Annaba, Skikda, Alger and Djurdjura Mts.). In the North it is known also from the coast (Bizerte, Ghar El Melh), from the plain of Mateur (Ichkeul National Park) and along the Gulf of Tunis (Nahli Park, Sidi Thabet, Tunis, Carthage). In the Cap Bon Peninsula the species is quite common along the coast (Nabeul, Oued el Abid, Sidi Erraiès, Kélibia, Dar Chichou, El Haouaria) but is also found inland, in Jebel Abderrahman. In Central Tunisia it occurs from the High Tell in the West (Le Kef, Tajerouine, Dugga, Nebeur), virtually from the whole Dorsale Ridge which stretches from Chambi and Kesra in the West to Jebel Bargou, Jebel el Fahs and Jebel Zaghouan in the East and further South from the plain of Kairouan (Sbikha, Thuburbo Majus) to the eastern coast in the so called Sahel (Sousse, Hergla, Békalta, Mahdia). In the South, the species was recorded from the mountains of Gafsa (Jebel Bou Ramli), the western Saharian platform (Midès) and from the Dahar Mts further east. It has been found as far south as Matmata and Tataouine.

**Map 1.**
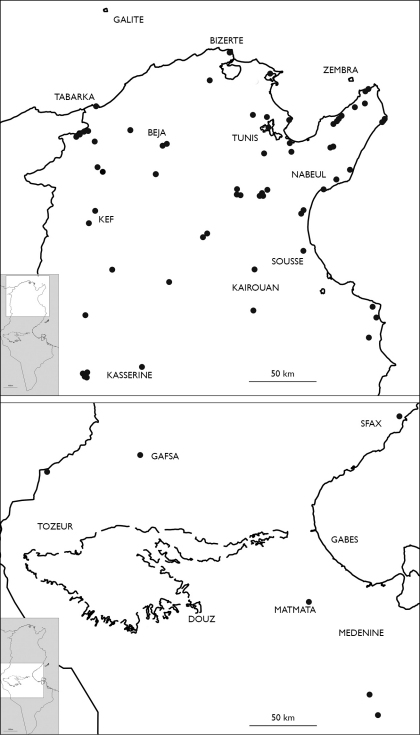
Distribution of Eupolybothrus nudicornis in Tunisia.

###### Altitudinal range in Tunisia.

Known from sea level up to approx. 1500 m. In Italy Eupolybothrus nudicornis has been recorded up to 2500 m altitude (Zapparoli 2006).

###### Chorotype.

W-Mediterranean, according to the chorotype classification of the W-Palearctic fauna proposed by Vigna Taglianti et al. (1993, 1999).

##### Eupolybothrus kahfi

Stoev & Akkari sp. n.

urn:lsid:zoobank.org:act:B9222D42-A69E-47EF-8ACE-7226398E489B

[Fig F3] Figs 3-4

###### Type material.

Holotype: adult ♂, North Tunisia, Zaghouan Governorate, Jebel Zaghouan, Gouffre (chasm) Sidi Bou Gabrine, 36°22.423'N, 10°06.328'E, alt. 642 m, under clay lump, 17.III.2008, P. Stoev leg. (NMNHS). Other material: 1 juv., same locality, date and collector, collected creeping on the wall at the endmost hall (NMNHS).

###### Diagnosis.

A species of Eupolybothrus with long antennae, approx. 90% length of body, composed of 68 articles; eyes composed of 18 ocelli; colour uniformly yellow-whitish; anterior margin of forcipular coxosternite with 7+7 teeth; TT 9, 11, 13 with posterior triangular projections; leg 15 56–57% length of body, with a single claw on pretarsus; prefemur of leg 15 with a long, conical dorso-median protuberance emerging from its posterior part and pointing posterior-dorsad; coxal pores generally large, round to ovoid; around 15–20 on legs 12 and 13 and about 20–24 on legs 14 and 15; posterior margin of male first genital sternite straight.

###### Description.

**Figure 3. F3:**
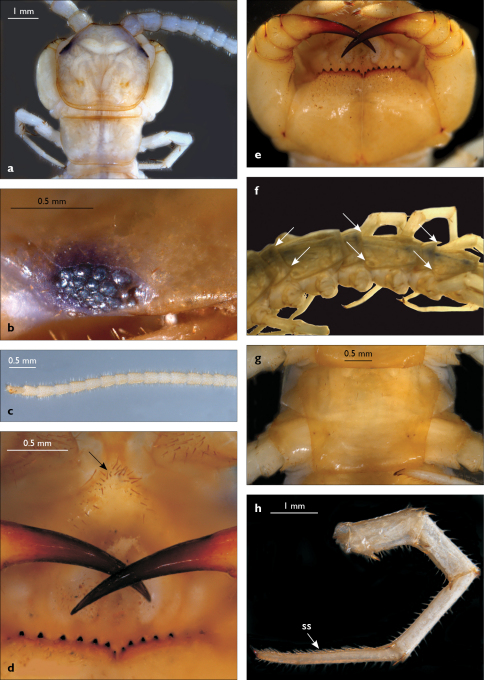
Eupolybothrus kahfi sp. n., male, holotype: a – cephalic plate; b – ocelli and Tömösváry’s organ; c – apical part of antenna; d – clypeus; e – forcipule; f – TT 8-14; g – sternite 7; h – leg 10. Figs 3e and f without scales. ss – serial setae. Posterior triangular projections on TT 9, 11 and 13 indicated by arrows (Fig. 3f), clypeal setae indicated by an arrow (Fig. 3d).

Holotype: Length (from anterior margin of cephalic plate to posterior margin of telson) approx. 30 mm; cephalic plate slightly broader than long (Fig. 3a); head 2.7 mm long, 3 mm wide; leg 15 aprox. 17 mm long, or 56–57% length of body. Colour generally uniformly yellow-whitish; only forcipular coxosternal teeth, posterior half of forcipular tarsungulum brown; anterior 1/3 of cephalic plate slightly darker yellowish; interrupted black line stretches along the midline of body and can be traced on all but last tergite.

Cephalic plate smooth, wider than T1 (Fig. 3a); a median notch contributing to biconvex anterior margin; marginal ridge with a distinct median thickening occupying almost 50% breadth of plate; posterior margin straight or slightly convex; central part of cephalic plate concave; transverse suture situated at about 1/3 of anterior edge; posterior limbs of transverse suture visible, connecting basal antennal article with anterior part of ocellar area; setae on cephalic plate very few, dispersed, without regular arrangement. Ocelli: 1+3,4,5,5; seriate ocelli greyish, oval to elliptical, in 4 rows: first seriate ocellus of the exteriormost row largest, ocelli of the middle two rows medium-sized, those of inferior row smallest; posterior ocellus as large as the first seriate ocellus. Tömösváry’s organ small, circular, situated on subtriangular sclerotisation immediately below the inferiormost row of seriate ocelli (Fig. 3b).

Left antenna long, approx. 27 mm, reaching or slightly surpassing posterior margin of T12 when folded backwards; 90% length of body, composed of 68 articles; right antenna damaged, composed of at least 34 articles; basal two articles enlarged (Fig. 3a), most articles longer than broad; last 12 articles more elongated than others; ultimate article about same length as penultimate (Fig. 3c). Basal two articles less setose than others, which are densely covered with trichoid setae.

Clypeus with a cluster of about 30–33 long to medium-sized setae situated at apex and near the lateral margin (Fig. 3d).

Forcipule (Fig. 3e): coxosternite subhexagonal, lateral margins feebly convex; anterior margin set off as a rim by furrow that is impressed behind all teeth; coxosternal teeth 7+7, inner tooth smaller than others, its apex well posterior to outer tooth; median diastema small, strongly narrowed by the inner teeth; intradental distance varying, generally increasing towards lateral teeth; porodont arising from a small node below the dental rim, situated posteriad to teeth and well laterad to lateralmost tooth; base of porodont as thick as adjacent tooth or slightly thinner; coxosternite densely setose anteriorly; setae generally long, in approximately 7–8 irregular rows; another row of long setae visible behind anterior margin. Forcipular trochanteroprefemur medially concave with a small subtriangular outgrowth emerging at its posterior part; distal part of tarsungulum about six times longer than proximal part, devoid of setae; forcipular prefemur, femur and tibia fringed with a row of setae (sparse and irregular on the posterior half of prefemoral part).

Tergites (Fig. 3f) generally wrinkled (less so on smaller tergites); TT 9, 11, 13 with well-developed posterior triangular projections, less so on T9; posterior margination lacking on all tergites, poorly visible on last two tergites and on the posterior angles of T1; T1 subtrapeziform, wider than T3, posterior margin transverse. Posterior margin of TT 8, 10, 12, 14 gently concave; posterior angles of TT 1, 2, 3, 4, 5 rounded; those of TT 6, 7, 8 right-angled; pointed on TT 10, 12, and less so also on T14; all tergites covered with sparse, thin but generally long setae, which increase in number towards posterior segments; posterior half of intermediate tergite covered with denser field of such setae.

Sternites smooth, subtrapeziform, with few sparse setae, mainly at lateral margins. Posterior margins straight, slightly convex only on sternites 1 and 15 (Fig. 3g).

**Figure 4.**
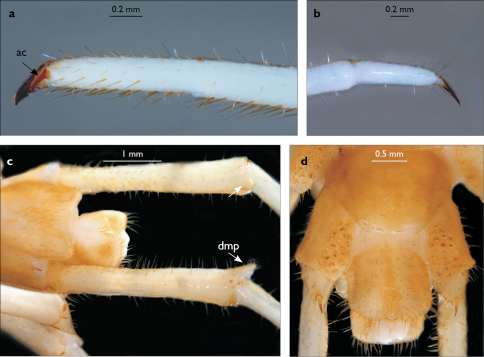
Eupolybothrus kahfi sp. n., male, holotype: a – tarsus 1, tarsus 2 and pretarsus of a midbody leg; b – tarsus 1, tarsus 2 and pretarsus of leg 15; c – prefemora of legs 15, dorso-lateral view; d – coxae and male first genital sternite. ac - accessory claw; dmp - dorso-median protuberance.

Legs: all legs generally elongated (Fig. 3h); legs 14 and 15 much longer than 1–12; leg 13 only slightly longer; leg 15 longest of all; maximal length of podomeres: coxa 1.3 mm, prefemur 3.2 mm, femur 3.2 mm, tibia 3.8 mm, tarsus 1 4.2 mm, tarsus 2 2.3 mm, pretarsus 0.4 mm. Tarsus 1 and tarsus 2 of legs 1–14 with two rows of ventral setae (Fig. 4a). Pretarsus of legs 1–14 with a large principal claw and smaller and thinner accessory claw emerging dorso-laterally; accessory claw half length of the principal claw. Pretarsus of leg 15 with a single claw (Fig. 4b). Leg 15 with secondary sexual modifications: prefemur with a long conical dorso-median protuberance emerging from its posterior part and pointing posterio-dorsad (Fig. 4c), its tip surmounted with a tuft of setae. Leg 14 without particular modifications.

Spinulation: as in Table 5.

Coxal pores: generally large, round to ovoid; 15–20 on legs 12–13 and about 20–24 on legs 14 and 15; pores separated by less than their own diameter, forming 3–4 irregular rows (Fig. 4d).

Male first genital sternite subquadrate (Fig. 4d), fringed with numerous long setae sparsely covering its whole surface, posterior margin not emarginated; gonopod small, hidden behind the edge of first genital sternite, with 8–10 long setae.

Juvenile: pale yellow-whitish, with 15 leg-pairs, most detached; antennae long, approx. 90% of body length, composed of 36–37 articles; ultimate article almost 2.5 times length of penultimate; 5 ocelli; forcipular coxosternite with 5+5 teeth, median diastema shallow, V-shaped; TT 9, 11, 13 with posterior triangular projections; coxal pores: 2,1,1,1.

**Table 5:** Eupolybothrus kahfi Stoev & Akkari, sp. n., male, holotype: spinulation of legs

Leg	Ventral					Dorsal				
	C	Tr	PFe	Fe	Ti	C	Tr	PFe	Fe	Ti
1	-	-	mp	amp	amp	-	-	amp	A	a
2	-	-	mp	amp	amp	-	-	amp	a-p	a
3	-	-	mp	amp	amp	-	-	amp	a-p	a-p
4	-	-	mp	amp	amp	-	-	amp	a-p	a-p
5	-	-	mp	amp	amp	-	-	amp	a-p	a-p
6	-	-	(a)mp	amp	amp	-	-	amp	a-p	a-p
7	-	-	(a)mp	amp	amp	-	-	amp	a-p	a-p
8	-	-	amp	amp	amp	-	-	amp	a-p	a-p
9	-	-	amp	amp	amp	-	-	amp	a-p	a-p
10	-	(m)	amp	amp	amp	-	-	amp	a-p	a-p
11	-	m	amp	amp	amp	-	-	amp	a-p	a-p
12	-	m	amp	amp	amp	-	-	amp	a-p	a-p
13	-	m	amp	amp	amp	-	-	amp	a-p	a-p
14	-	m	amp	amp	a	a	-	am	a-p	a-p
15	a	m	amp	am	a	a	-	am	P	-

###### Origin of name.

derives from the Arabic word kahf (ﻒﮫﮐ) meaning ‘cave’, and kahfi denotes ‘living in cave’.

###### Habitat.

Eupolybothrus kahfi occurs in a chasm of approximately 30 m depth which after descending continues as a narrow horizontal gallery ending in a small hall. The total length of the cave is approximately 50 m. There are just a few humid spots on the floor, with almost no organic substance. The juvenile specimen was collected creeping on the wall at the end hall, while the adult was found under a lump of clay, approximately one meter below the place of descent. In the cave Eupolybothrus kahfi co-occurs with troglomorphic isopods, spiders of the genus Meta C.L. Koch, 1836, pseudoscorpions of the genus Roncus L. Koch, 1873, harvestmen, troglobitic diplurans, trichopterans and gastropods.

### Morphology

With very few exceptions, Tunisian specimens of Eupolybothrus nudicornis fit the morphological diagnosis of Eupolybothrus nudicornis elongatus well. Eason (1972a) reported on specimens with intermediate characters from Constantine, Algeria, and wrote “…it seems that the characters separating elongatus and impressus are unstable”, and “..…in spite of the intermediate examples from Constantine, it seems advisable to retain, for the time being, the distinction between elongatus and impressus …but they should be regarded as only subspecifically distinct.” Most of the specimens we studied lack triangular projections on T9, though sometimes they were angulated or only slightly projecting behind the rear margin (specimens from Ichkeul National Park, ZMUC). All specimens except for one adult female from Jebel Chambi and one adult female from near Oued El Abid village lacked spines on tarsi of legs 15. Only two specimens out of hundreds possessed tarsal spines. Likewise, the shape of tergite 9 seems to be also infrapopulationally variable. The specimens from Spain (Granada) in Meinert’s collection (ZMUC), which were studied by E.H. Eason in 1980, all lack triangular projections and tarsal spines and should therefore be attributed to Eupolybothrus nudicornis elongatus even if geographically this area is situated within the range of the nominate form. The general colour of the body also varies considerably among the populations, from uniformly dark brown in e.g., the Ichkeul specimens, to uniformly castaneous and dark yellowish-brownish in most of the other examined specimens. Some specimens (e.g., those from near Zahret Médine and Bulla Regia, NMNHS) possess a dark middorsal band.

### Molecular data

**Figure 5.**
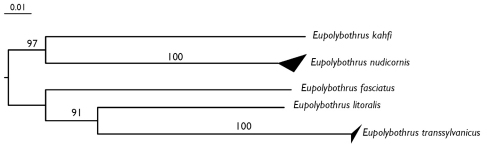
Neighbor joining tree (K2P) of 5 species of Eupolybothrus based on the COI 5’ ‘barcoding fragment’. Bootstrap support values are shown on the branches. The upper and lower sides of the triangle represent respectively the maximum and minimum of genetic distances within the species.

In order to confirm the delineation of the new species Eupolybothrus kahfi, we used DNA barcoding to bring genetic support to the morphological observations. The COI barcodes examined from 11 specimens (Table 6) among 5 species of Eupolybothrus exhibited a 20.8% mean value for interspecific divergences (Table 7). The lowest value was 16.61% between Eupolybothrus transsylvanicus and Eupolybothrus litoralis, and the highest was 23.98% between Eupolybothrus transsylvanicus and Eupolybothrus nudicornis. By contrast, for the two species for which we were able to measure it, we observed a low infraspecific value, 1.14% for Eupolybothrus nudicornis (sampled from three different populations in Tunisia; see Table 6) and 0.3% for Eupolybothrus transsylvanicus The neighbor joining tree built from this dataset shows the clear separations between the different barcode clusters corresponding to the different species (Fig. 5).

**Table 6:** Specimens sequenced for COI and their BOLD and GenBank accession numbers.

Species	Locality	GenBank accession number	BOLD accession number
Eupolybothrus litoralis	Turkey, Afyon Prov., near village of Akoren	HM065035	NMNHS-PES-00062
Eupolybothrus nudicornis	Tunisia, Cap Bon Peninsula, 20 km from El Haouaria	HM065036	NMNHS-PES-00077
Eupolybothrus nudicornis	Tunisia, Cap Bon Peninsula, 7 km from Menzel Bou Zelfa	HM065037	NMNHS-PES-00079
Eupolybothrus nudicornis	Tunisia, Nabeul, plage El Mansoura	HM065038	NMNHS-PES-00053
Eupolybothrus nudicornis	Tunisia, Nabeul, plage El Mansoura	HM065039	NMNHS-PES-00052
Eupolybothrus nudicornis	Tunisia, Jebel Zaghouan, surroundings of the marabout Sidi Bou Gabrine	HM065040	NMNHS-PES-00045
Eupolybothrus nudicornis	Tunisia, Jebel Zaghouan, surroundings of the marabout Sidi Bou Gabrine	HM065041	NMNHS-PES-00044
Eupolybothrus kahfi	Tunisia, Jebel Zaghouan, Gouffre Sidi Bou Gabrine	HM065042	NMNHS-PES-00046
Eupolybothrus transsylvanicus	Bulgaria, Shumen City	HM065043	NMNHS-PES-00066
Eupolybothrus transsylvanicus	Bulgaria, Shumen City	HM065044	NMNHS-PES-00065
Eupolybothrus fasciatus	Italy, Fogliano Mt, near Viterbo, Lazio	AY214420	--------------------------

**Table 7:** Genetic distances between species within Eupolybothrus (K2P-pairwise).

		1	2	3	4
1	Eupolybothrus nudicornis				
2	Eupolybothrus litoralis	21.4			
3	Eupolybothrus transsylvanicus	23.98	16.61		
4	Eupolybothrus kahfi	19.19	22.28	23.46	
5	Eupolybothrus fasciatus	21.47	18.15	20.7	21.56

## Discussion

### Taxonomy.

Eupolybothrus kahfi’s nearest neighbor is Eupolybothrus nudicornis with 19.19% divergence. This interspecific value is consistent with the distances observed among the other examined species of the genus. Thus it contributes genetic support to the delineation of this new species which appears as a well individualized mitochondrial lineage. Unfortunately we do not have more specimens of Eupolybothrus kahfi’s but we can reasonably expect low values for intraspecific variation. The results for Eupolybothrus nudicornis and Eupolybothrus transsylvanicus show low intraspecific divergences for COI by comparison to the interspecific divergences, confirming the ‘barcoding gap’ described by Hebert et al. (2003) as a general principle of DNA barcoding. These preliminary results support the use of DNA barcoding for a clear discrimination of closely related species within the genus Eupolybothrus. In addition to the molecular support, Eupolybothrus kahfi’s is very well characterized morphologically, having a long, conical dorso-median protuberance at the prefemur of leg 15, a unique trait within the genus. This is of importance for discussions of cryptic diversity because it will permit pointing to the right genetic entity/COI cluster as the bearer of the species name, and then to assign a new name to the other sibling species. Although it may be possible to get the sequence from museum material for this purpose (Hausmann et al. 2009), it is easier and cheaper to barcode the fresh holotype at the same time it is described.

Jeekel (1967) assigned Eupolybothrus nudicornis and allied taxa to the subgenus Allopolybothrus Verhoeff, 1907, characterised by the following set of morphological characters: absence of VCm spine and presence of VCa spine on leg 15, single pretarsus of leg 15, posterior triangular projections on TT 9, 11, 13 (often reduced on T 9), spinulation of leg 15: 0, 1, 3–4, 1–2, 0–1, (1), male gonopods short, single-segmented. In the same publication he wrote “… the practical value of these subgenera is doubtful” and indeed all those characters are found also in species from other subgenera and thus are of very little value for establishing the phylogenetic relationships among the species. Most of the subgenera of Eupolybothrus comprise only a limited number of species, some of which are poorly described and known from a single specimen only. It is beyond the scope of this publication to revise the whole genus, nevertheless we would like to point out that the currently accepted subgeneric division of Eupolybothrus is outdated and will most likely be altered once several poorly known taxa are revised and contemporary phylogenetic methods are applied.

No taxonomically significant differences were found between the syntype specimens of Eupolybothrus cloudsley-thompsoni and Eupolybothrus nudicornis, which confirms Zapparoli’s (1985) suspicion that both might be identical. The examined specimens lack posterior triangular projections on T9 and tarsal spines which characterize the Tunisian populations. Instead of trying to distinguish the new species from the other North African congeners known at that time, Turk (1955) compared the new species with the morphologically and geographically quite distant Eupolybothrus segregans Chamberlin, 1952 and Eupolybothrus praecursor (Attems, 1902) from Turkey and Lebanon, respectively, both currently considered synonyms of Eupolybothrus litoralis (cf. Zapparoli 1991, 1995b). He also wrongly attributed one juvenile Lithobius castaneus to the syntype series of Eupolybothrus cloudsley-thompsoni and failed to illustrate the porodonts. Turk’s species was improperly justified, and we regard Eupolybothrus cloudsley-thompsoni to be conspecific with Eupolybothrus nudicornis.

Matic et al. (1992) described two new Italian species of Eupolybothrus, Eupolybothrus osellai and Eupolybothrus ruffoi from the Cozian Alps and Apuan Alps, respectively. Both species were very vaguely diagnosed and described, as no comparison with other congeners was made. They are morphologically similar to Eupolybothrus nudicornis, and except for some minor differences in the spinulation there are no sound traits that allow separation from the latter. The possible synonymy with Eupolybothrus nudicornis has aleady been suspected (Minelli 2006). The question whether Eupolybothrus nudicornis represents a single polymorphic species or a species-complex comprising cryptic (sub-)species is also beyond the scope of this paper and requires examination of additional material from Europe and extension of molecular sampling. A fact of interest is the absence of Eupolybothrus nudicornis from the Balearic Islands (see e.g., Sammler et al. 2006), and its extreme rarity in Spain, which can hardly be explained as an artifact of collecting activities in these regions.

### Post-embryonic development.

Information on the post-embryonic development of species of Eupolybothrus is generally poor, as more comprehensive studies have been published only for Eupolybothrus nudicornis (Meinert 1872, Daas et al. 1996), Eupolybothrus grossipes and Eupolybothrus litoralis (Eason 1970), Eupolybothrus dolops (Zapparoli 1998) and Eupolybothrus transsylvanicus (Mitić and Tomić 2008). The number of post-larval stadia was found to be species-specific but could also vary intraspecifically in the different parts of the species’ range (Andersson 1981). Thus, Eason (1970) distinguished and described six post-larval stadia in Eupolybothrus grossipes, which corresponds to the number of stadia found also in Eupolybothrus transsylvanicus (Mitić and Tomić 2008). Daas et al. (1996) also reported six post-larval stadia for the Algerian populations of Eupolybothrus nudicornis (sub elongatus). Murakami (1958) reported eight post-larval stadia (including matures) in Bothropolys rugosus (Meinert, 1872) (sub Bothropolys asperatus). Our data on postembryonic development (Table 4) agree with those given by Meinert (1872) (Table 2), except for the higher number of forcipular coxosternal teeth in the larval stadia. Compared with the data of Daas et al. (1996) (Table 3), there are some differences; for example, our data show higher number of antennomeres in larval stadia III and IV and less in PLI. This could be due to geographical variation.

### Distribution.

**Map 2.**
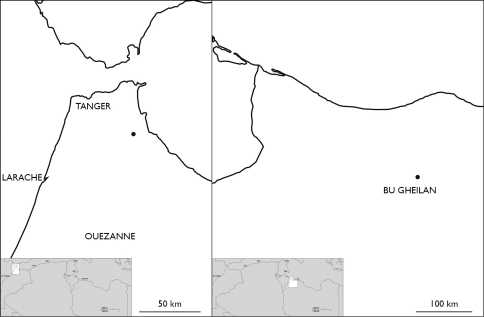
Localities of Eupolybothrus nudicornis in Libya and Morocco.

**Map 3.**
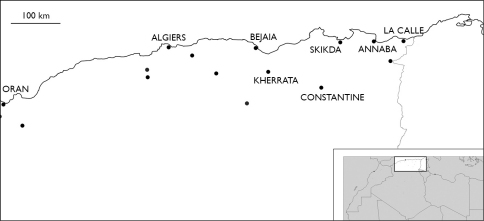
Distribution of Eupolybothrus nudicornis in Algeria.

Eupolybothrus nudicornis is distributed throughout the whole of Maghreb, although from Morocco and Libya it is so far known only from single localities – near Bab Berred (Tetouan) (Zapparoli 1985) and in Bu Gheilan (Manfredi 1939), respectively (Map 2). The majority of records come from North Algeria (Map 3) and Tunisia (Map 1). The species distribution in North Africa covers an area of approx. 894 000 sq. km, or a distance of 1,720 km East-West and 520 km North-South. The species occurs also on Malta and Gozo (Zapparoli et al. 2004). In Europe it is known from France (Basses Alpes, Alpes Maritimes, Corsica) and Italy (Sardinia and circum-Sardinian islands, Ponziane Isl. [Santo Stefano Is.], Ischia Is., Sicily, Eolie [Filicudi, Lipari, Salina, Vulcano], Egadi [Favignana, Levanzo], Ustica, Lampedusa and Pantelleria Islands. In Spain it is hitherto known only from Granada (Meinert 1872) and Linares (Attems 1952).

**Figure 6.**
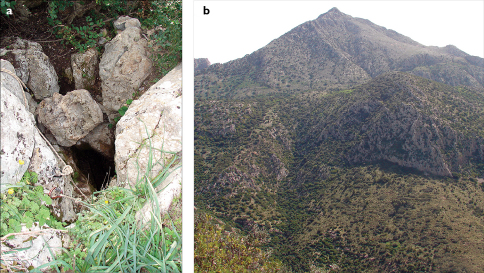
a – A view of the entrance of cave Sidi Bou Gabrine, Jebel Zaghouan. b – A view of Jebel Zaghouan, Zaghouan Governorate, NE Tunisia.

**Map 4.**
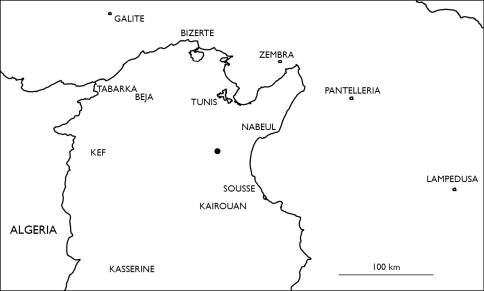
Locality of Eupolybothrus kahfi in Tunisia.

Eupolybothrus kahfi is known only from its type locality, the cave Sidi Bou Gabrine (Fig. 6a). The cave is situated in the limestone massif Jebel Zaghouan (Fig. 6b) at a distance of approximately 500 m from the marabout Sidi Bou Gabrine (Map 4). The southwestern part of the mountain is composed of Jurassic limestone strata of mostly Sinemurian to Tithonian age (Schlüter 2006). There are at least 30 caves in Jebel Zaghouan and around 20 in the neighbouring mountains (Mohammed Tiouiri pers. comm.) and it is very likely that Eupolybothrus kahfi will be found elsewhere once more profound biospeleological investigations are carried out.

### Habitats.

In Tunisia Eupolybothrus nudicornis is recorded from coniferous and broad-leaf woods of different composition and dominant structure: 1) oak forests dominated by Quercus suber and Erica arborea; 2) coniferous forests dominated by Pinus halepensis; 3) mixed forests with Pinus halepensis, Quercus ilex and Stipa tenacissima; mixed forests with Olea europaea and Pistacia lentiscus; mixed woods with Eucalyptus and Thuja; 4) Olea europaea orchards. Eupolybothrus nudicornis has been found also in open habitats such as meadows with scattered vegetation, coastal slopes with planted vegetation, rocky terrains overgrown with shrubs not far from the sea (approx. 10–50 m from the water line), coastal sandy habitats with very scattered halophilous vegetation, maquis, arid rocky slopes with shrubs and stones, deserted rocky plains with Opuntia and sparse palm trees, suburban and urban habitats, and ruins.

Minelli and Iovane (1987) consider it a “fairly euryecious” species in Italy, where it often inhabits woods (Aquifolium-Fagetum, Quercus cerris, Quercus ilex, Castanea, Ostrya), but also open habitats (Plantago cupanii, Calycotome, Genisto-Potentilletum, Cynosuro-Leontodontetum), occassionaly found also on dunes, gardens, Olea stands, but seldom in caves. According to Zapparoli (2006), in the Central Apennines the species is most common in pastures, grasslands and open or shrub montane habitats above 900–1000 m. It occurs also in the Fagus-shrub ecotone, in garigues and calanques, seldom in Quercus cerris or Ostrya woods, olive groves, Pinus spp. reforestations, urban and suburban gardens and parks. On Malta and Gozo, Eupolybothrus nudicornis is known from a range of habitats including widien (valleys carrying water only during the wet season), leaf litter under Acacia and Ceratonia siliqua trees, in garique, coastal vegetation, gardens and urbanised areas (Zapparoli et al. 2004). On Panetelleria Island it also inhabits woods of Quercus ilex (Zapparoli 1995a). In Sardinia it is known from sea level up to 1800 m, in oakwoods (Quercus ilex), pine and Eucalyptus plantations, as well as in garrigue and agricultural habitats (walnut orchards); also recorded from caves and in endogeous habitat (Zapparoli 2009).

Unlike Eupolybothrus nudicornis, Eupolybothrus kahfi is known only from a cave showing traits of adaptation for life underground (e.g., long legs and antennae, pale coloration). It is worth mentioning that still very little is known about the cavernicolous lithobiomorphs in North Africa. Cave-dwelling lithobiomorphs are hitherto unknown from Libya and Egypt. Only three species have hitherto been recorded from caves in Algeria and Morocco, these all being members of Lithobius Leach, 1814 (cf. Boutin et al. 2001, Decu et al. 2001). Only Lithobius chikerensis Verhoeff, 1936 shows troglomorphic traits (long antennae, large Tömösváry’s organ, reduced ocelli) and was categorised as a troglophile (Zapparoli 1984). It is known from the Ben Add cave in Oran, Algeria and from the caves Daya Chiker, Friouat and Ras el Ma in Taza province, Morocco (Brolemann 1931b, Verhoeff 1936, Manfredi 1956, Matic 1967, Zapparoli 1984). The other two species, Lithobius crassipes L. Koch, 1862 and Lithobius dieuzeidei Brolemann, 1931 are occasional cave-dwellers and represent trogloxenes at most (Zapparoli 1984).

## Identification key to the species of Eupolybothrus

**Table:** 

1 (10)	Ocelli (Figs k-1-2) or posterior triangular projections on tergites absent (Fig. k-3)	2
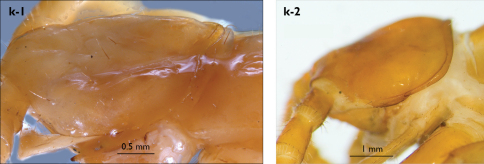		
2 (5)	Ocelli absent (Fig. k-1), posterior triangular projections at least on TT 9, 11, 13 (Fig. k-4)	3
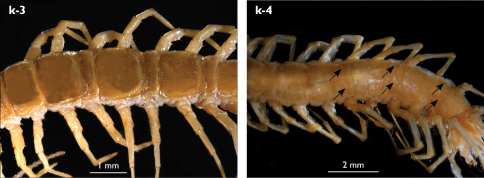		
3 (4)	Forcipular coxosternite with 9+9-12+12 teeth (Fig. k-5), forcipular trochanteroprefemur unmodified, 15VCa and 15 DCa spines present, pretarsus of leg 15 with a single claw (Fig. k-6)	Eupolybothrus obrovensis (caves in Italy, Slovenia, Croatia)
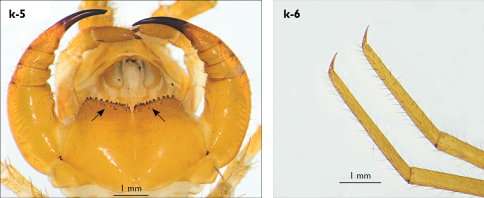		
4 (3)	Forcipular coxosternite with 13-14 teeth (Fig. k-7), forcipular trochanteroprefemur strongly swollen, 15VCa and 15 DCa spines absent, pretarsus of leg 15 with a principal claw and posterior accessory claw (Fig. k-8)	Eupolybothrus andreevi (cave in Bulgaria)
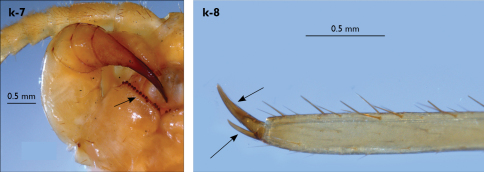		
5 (2)	Ocelli present (Fig. k-9), all tergites without posterior triangular projections (Fig. k-10)	6
6 (7)	Pretarsus of leg 15 with accessory claw	Eupolybothrus verrucosus (Moldavia)
7 (6)	Pretarsus of leg 15 without accessory claw	8
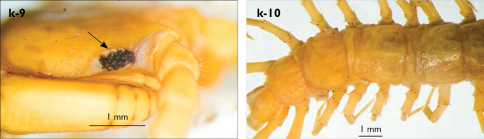		
8 (9)	T1 much broader than head (Fig. k-11), deeply concave posteriorly; forcipular trochanteroprefemur with a dorso-lateral knob (Fig. k-12)	Eupolybothrus dolops (Greece)
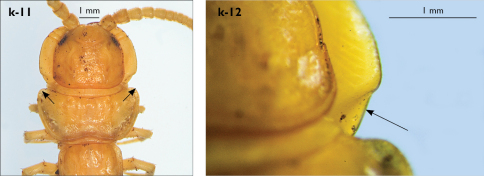		
9 (8)	T1 as broad or slightly broader than head (Fig. k-13), transverse posteriorly; forcipular trochanteroprefemur without a knob	Eupolybothrus werneri (Greece, Albania)
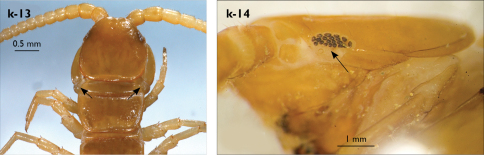		
10 (1)	Ocelli present (Fig. k-14); posterior triangular projections present at least on TT 11 and 13 (Fig. k-15)	11
11 (20)	VCm spine present on leg 15 (Fig. k-16)	12
12 (13)	Six ill-defined, feebly pigmented ocelli in adults	Eupolybothrus leostygis (caves in Bosnia and Herzegovina, Croatia)
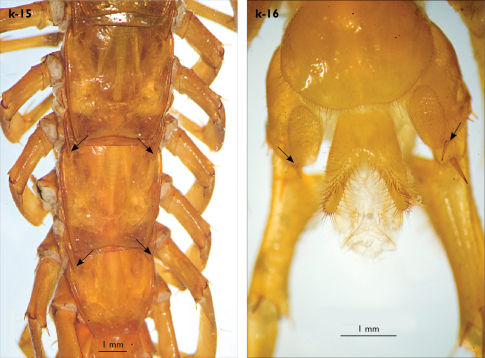		
13 (12)	10-25 pigmented ocelli in adults (Fig. k-17)	14
14 (19)	Male leg 15 with a large rounded knob proximate of the middle of the caudal side of the prefemur (Fig. k-18)	15
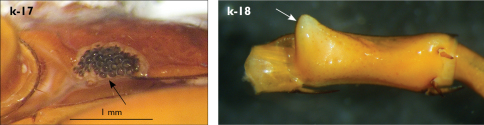		
15 (18)	Antennae composed of 74-83 antennal articles	16
16 (17)	Prefemoral knob simple (Fig. k-19)	Eupolybothrus acherontis (here possibly also belong the poorly known acherontis wardaranus from FYR Macedonia and Eupolybothrus stygis from Ilijna cave in Bosnia and Herzegovina) (FYR of Macedonia, Bosnia and Herzegovina)
17 (16)	Prefemoral knob apically incised forming two rounded processes densely covered with trichoid setae (Fig. k-20)	Eupolybothrus excellens (Italy)
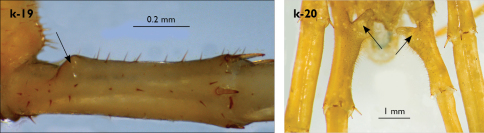		
18 (15)	Antennae composed of 50-60 antennal articles	Eupolybothrus caesar (here possibly also the poorly known Eupolybothrus spiniger from Bosnia and Herzegovina) (Albania, Greece, FYR of Macedonia, Bosnia and Herzegovina)
19 (14)	Male leg 15 without prefemoral knob (Fig. k-21)	Eupolybothrus tabularum (Italy)
20 (11)	VCm spine absent (Fig. k-22)	21
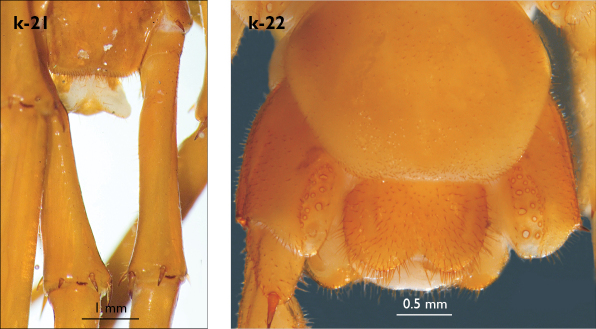		
21 (28)	Posterior triangular projections present on TT 9, 11, 13 or 11, 13 (Fig. k-23)	22
22 (25)	Male gonopods long (Fig. k-24)	23
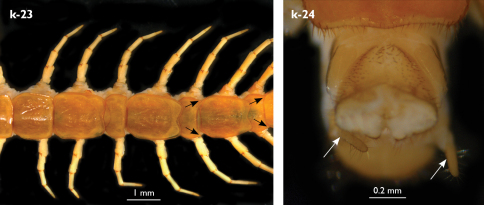		
23 (24)	17 ocelli, last 15 antennal articles shorter, only the ultimate article being 3 times longer than broad, others 2 times at most	Eupolybothrus zeus(here also probably Eupolybothrus sketi from FYR Macedonia) (Greece)
24 (23)	Six-seven ocelli; last 15 articles of antennae elongated, 3 times longer than broad	Eupolybothrus macedonicus (Greece)
25 (22)	Male gonopods short (Fig. k-25)	26
26 (27)	Leg 15 approx. 30% length of body; prefemur of leg 15 in male moderately enlarged with two paramedian sulci not extending to posterior margin; posterior part of prefemur swollen dorso-medially (Fig. k-26); posterior margin of male first genital sternite emarginated (Fig. k-22)	Eupolybothrus nudicornis (North Africa from Morocco to Libya, Spain, France, Italy, Malta)
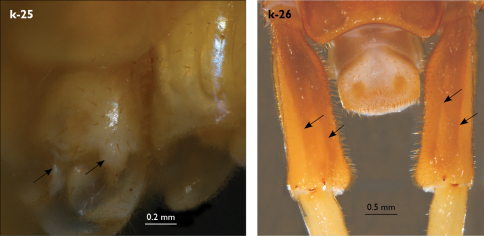		
27 (26)	Leg 15 approx. 60% length of body; male prefemur 15 with a long, conical dorso-median protuberance (Fig. k-27); posterior margin of male first genital sternite not emarginated (Fig. k-28)	Eupolybothrus kahfi (cave in Tunisia)
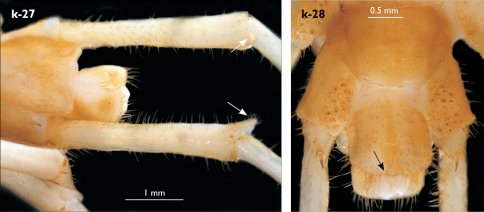		
28 (21)	Posterior triangular projections present on TT 6, 7, 9, 11, 13 or 7, 9, 11, 13 (Fig. k-29)	29
29 (32)	Male gonopods short, two-jointed (Fig. k-25), VCa spine on leg 15 present (Fig. k-30), pretarsus of leg 15 without accessory claw	30
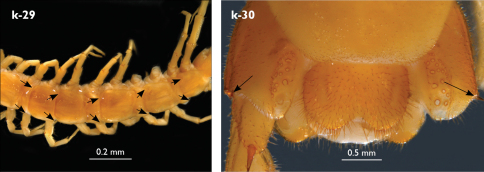		
30 (31)	Prefemur of male leg 15 inflated and strongly expanded medially just proximad of the middle, the protuberance densely covered with setae (Fig. k-31); a densely setose circular dorsomedial area covering almost ? of prefemoral breadth in the position of DPFep spine, which is absent (Fig. k-31)	Eupolybothrus imperialis (Italy)
31 (30)	Prefemur of male leg 15 without such protuberance, evenly expanded along its whole length (Fig. k-32); circular area smaller, covering 1/3 of prefemoral breadth at most (Fig. k-32)	Eupolybothrus herzegowinensis (Albania, Bosnia and Herzegovina, Montenegro)
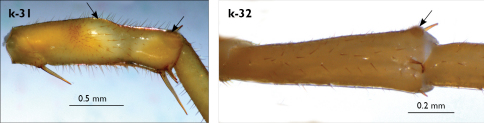		
32 (29)	Male gonopods long (unknown in Eupolybothrus valkanovi) (Fig. k-24), VCa spine on leg 15 usually absent (rarely only in Eupolybothrus litoralis), pretarsus of leg 15 with accessory claw (Fig. k-33)	33
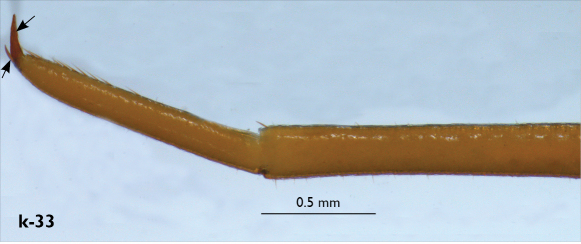		
33 (34)	Antennae long, composed of 58-90 articles (Fig. k-34); leg 15 almost as long as body, densely covered with long setae 4-4.5 times diameter of article (Fig. k-35)	Eupolybothrus gloriastygis (caves in Bosnia and Herzegovina, Croatia, Montenegro; the record of Stoev (2001c) from two caves in Bulgaria is erroneous and probably refers to morphologically closely related but different (new) species)
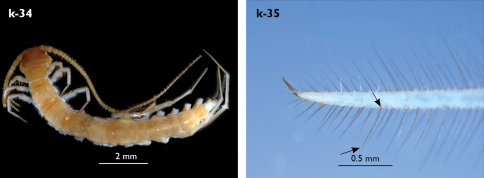		
34 (33)	Antennae and legs shorter (Fig. k-36), setae on leg 15 shorter (Fig. k-37)	35
35 (42)	Pretarsus of leg 15 with accessory apical claw (Fig. k-33)	36
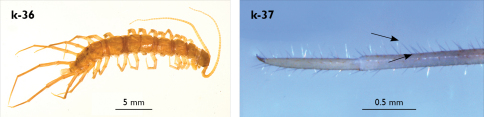		
36 (39)	Seriate setae on tarsus 2 of leg 15 absent (Fig. k-38)	37
37 (38)	Antennae and leg-pair 15 elongated, about 3/4 body length (Fig. k-36); femur of male leg 15 without basal pit (Fig. k-39)	Eupolybothrus longicornis (France, Italy)
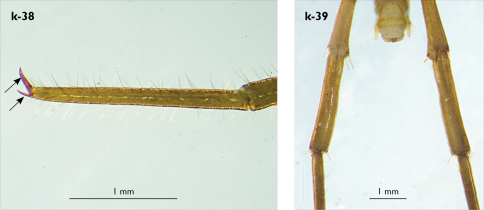		
38 (37)	Antennae and leg-pair 15 about half body length; femur of male leg 15 with an extensive and deep basal pit (Fig. k-40)	Eupolybothrus litoralis (southern Balkans, Near East)
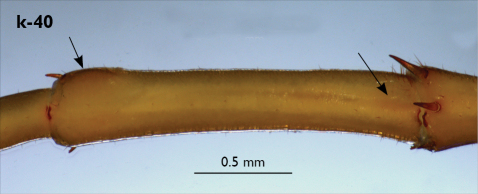		
39 (36)	Seriate setae on tarsus 2 of leg 15 present (Fig. k-33)	40
40 (41)	Basal pit of femur in male leg 15 extensive and deep; internal dorsal sulcus of femur in male leg 15 not extending to margin of pore-free area which bears a prominent globular swelling (Fig. k-41)	Eupolybothrus fasciatus (Italy, France, uncertain records from the Balkan Peninsula)
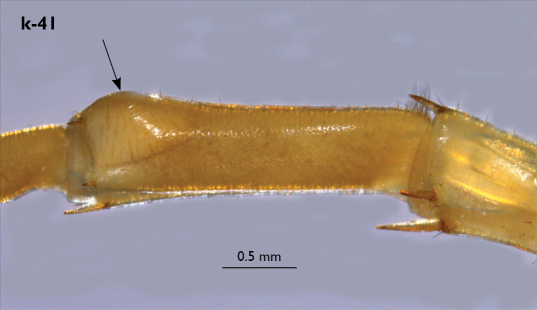		
41 (40)	Basal pit of femur in male leg 15 small and shallow; internal dorsal sulcus of femur in male leg 15 extending to margin of pore-free area which is not swollen (Fig. k-42)	Eupolybothrus grossipes (France, Italy, Switzerland, Austria, Slovenia, Germany, Serbia?, Romania?)
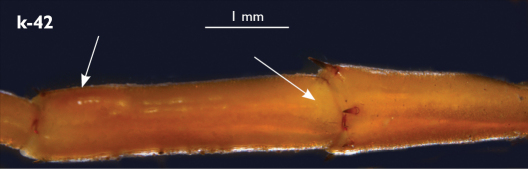		
42 (35)	Pretarsus of leg 15 without accessory apical claw (Fig. k-43)	43
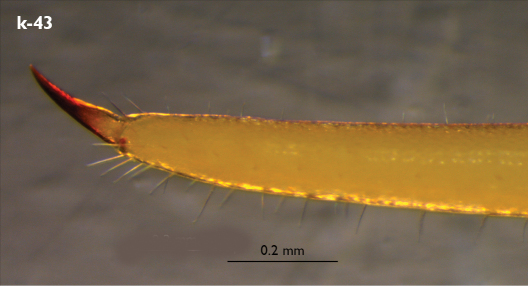		
43 (44)	Pretarsus of leg 15 with a small accessory claw emerging basally to the principal claw (Fig. k-44), adults: 16-25 mm; T6 without posterior triangular projections (Fig. k-45)	Eupolybothrus tridentinus (Albania, Austria, Bosnia and Herzegovina, Bulgaria, Croatia, Czech Republic, Germany, Hungary, Italy, Romania, Serbia, Montenegro, Slovenia, Switzerland)
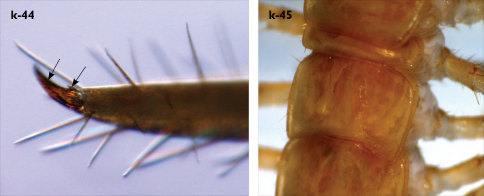		
44 (43)	Pretarsus of leg 15 without accessory basal claw (Fig. k-43), body length of adults more than 30 mm; T6 with broad posterior triangular projections (Fig. k-46)	Eupolybothrus transsylvanicus (here also probably Eupolybothrus valkanovi known from a single female) (Bosnia and Herzegovina, Bulgaria, Croatia, Greece, Hungary, Romania, Serbia, Montenegro)
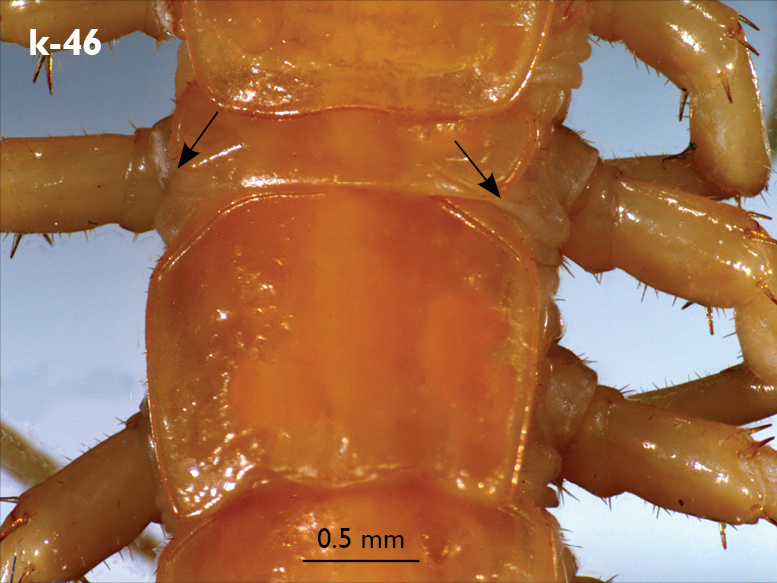		

## Supplementary Material

XML Treatment for Eupolybothrus

## Appendix A.

Locality dataset for all published and new records of Eupolybothrus nudicornis and Eupolybothrus kahfi in North Africa. File format: Microsoft Excel (1997-2003). doi:10.3897/zookeys.50.504-app.A

## Appendix B.

Character dataset for larval and post-larval stadia of Eupolybothrus nudicornis based on published (Meinert 1872, Silvestri 1896, Daas et al. 1996) and new data. Post larval stadia of the new material defined after Daas et al. 1996. File format: Microsoft Excel (1997-2003). doi:10.3897/zookeys.50.504-app.B

## Appendix C.

BOLD dataset comprising the COI registration numbers of sequenced Eupolybothrus specimens, COI-5P sequence length and locality data. File format: Microsoft Excel (1997-2003). doi:10.3897/zookeys.50.504-app.C

## Appendix D.

MorphBank dataset of all published figures comprising figure number, species name, locality data, body part and additional metadata. File format: Microsoft Excel (1997-2003). doi:10.3897/zookeys.50.504-app.D

## Appendix E.

GoogleEarth (http://earth.google.com) interactive map displaying the distribution of Eupolybothrus nudicornis and Eupolybothrus kahfi in North Africa. File format: KML (Keyhole Markup Language) version 2.1 for GoogleEarth. doi:10.3897/zookeys.50.504-app.E

## Appendix F.

Interactive key for identification of all currently valid species of Eupolybothrus made with Delta software. doi:10.3897/zookeys.50.504-app.F

Copyright notice: All datasets are published under the terms of the Creative Commons Attribution License, which permits unrestricted use, distribution, and reproduction in any medium, provided that the original author and source are credited.
